# Predicting the Global Potential Distribution of Four Endangered *Panax* Species in Middle-and Low-Latitude Regions of China by the Geographic Information System for Global Medicinal Plants (GMPGIS)

**DOI:** 10.3390/molecules22101630

**Published:** 2017-09-28

**Authors:** Zhixia Du, Jie Wu, Xiangxiao Meng, Jinhua Li, Linfang Huang

**Affiliations:** 1 Institute of Medicinal Plant Development (IMPLAD), Chinese Academy of Medical Sciences (CAMS), Peking Union Medical College (PUMC), Beijing 100193, China; zxdu712@126.com (Z.D.); jinhuali108@126.com (J.L.); 2 Institute of Chinese Materia Medica, China Academy of Chinese Medical Sciences, Beijing 100700, China; jwu1986@icmm.ac.cn (J.W.); xxmeng@icmm.ac.cn (X.M.)

**Keywords:** *Panax*, global potential distribution, geographic information system for global medicinal plants (GMPGIS)

## Abstract

Global biodiversity is strongly influenced by the decrease in endangered biological species. Predicting the distribution of endangered medicinal plants is necessary for resource conservation. A spatial distribution model—geographic information system for global medicinal plants (GMPGIS)—is used to predict the global potential suitable distribution of four endangered *Panax* species, including *Panax japonicas (T. Nees) C. A. Meyer* and *Panax japonicas var. major (Burkill) C. Y. Wu & K. M. Feng* distributed in low- and middle-latitude, *Panax zingiberensis C. Y. Wu & K. M. Feng* and *Panax stipuleanatus C. T. Tsai & K. M. Feng* in low-latitude regions of China based on seven bioclimatic variables and 600 occurrence points. Results indicate that areas of *P. japonicus* and *P. japonicus* *var. major* are 266.29 × 10^5^ and 77.5 × 10^5^ km^2^, respectively, which are mainly distributed in China and America. By contrast, the areas of *P. zingiberensis* and *P. stipuleanatus* are 5.09 × 10^5^ and 2.05 × 10^5^ km^2^, respectively, which are mainly distributed in Brazil and China. *P. japonicus* has the widest distribution among the four species. The data also indicate that the mean temperature of coldest quarter is the most critical factor. This scientific prediction can be used as reference for resource conservation of endangered plants and as a guide to search for endangered species in previously unknown areas.

## 1. Introduction

*Panax (Araliaceae)* species are the important medicinal resources in the world. *Panax ginseng* and *Panax quinquefolium* are distributed at high-latitude regions. By contrast, some endangered *Panax* species such as *P. japonicus* and *P. japonicus var. major* found in middle- and low-latitude regions, have been recorded in the Chinese Pharmacopoeia. *P. japonicus* is known as “the king of herbs” in Chinese folk, and has been included in the Japanese Pharmacopoeia as a traditional Japanese medicine. *P. zingiberensis* and *P. stipuleanatus* distributed in low-latitude regions are also used widely as traditional ethnic medicines. *Panax* species are popular due to their potential medicinal properties [1,2,3,4], such as anti-fatigue, anti-tumor, anti-thrombotic, anti-inflammatory, anti-oxidative, and immune-enhancing effects and thus have a substantial market demand worldwide [5,6,7]. However, their resources are gradually declining due to excessive harvesting and lack of environmental protection. Therefore, predicting the distribution of plants is necessary for their conservation [8].

With the development of network technology, a distribution prediction model has become one of the common methods in biodiversity conservation, such as MaxEnt (Maximum Entropy), Random Forset [9,10,11,12,13], GMPGIS (the geographic information system for global medicinal plants) [14,15,16]. The GMPGIS selected seven key ecological factors (especially related to its growth and accumulation of secondary metabolism) for medicinal plants according to biological characteristics of medicinal plants, statistics, ecology, botany, and related literatures, and experience. In this research, the global potential distribution of four endangered *Panax* species found in middle- and low-latitude regions was predicted by using GMPGIS. The GMPGIS model has been created by the Institute of Chinese Materia Medica in order to predict the distribution of medicinal plants [17] using environment databases such as WorldClim, CliMond, and HWSD. This model has been used for introduction and conservation of *Panax ginseng C. A. Mey* and *Panax notoginseng (Burk.) F. H. Chen* [18]. By combining the climate and soil factors to explore areas that have the most similar ecological factors, we can determine the suitable environment for medicinal plants to scientifically protect and cultivate the endangered plants. MaxEnt is also widely used to predict species distribution [19,20,21,22,23,24,25,26,27,28]. Potential suitable distribution plays an important role in resource protection and cultivation of endangered plants. We studied and compared the differences between GMPGIS and MaxEnt and found that GMPGIS shows higher precision than MaxEnt. In this research, GMPGIS was used to analyze the plant habitats, whereas MaxEnt was used for supplementary information.

We used GMPGIS to scientifically predict the global distribution of four endangered *Panax* species based on the climate and soil factors. The global potential suitable distributions were mapped by excluding the unsuitable areas, such as lakes, rivers, and cities. This research aims to predict the global potential suitable distribution of four endangered *Panax* species and provide a scientific reference for protecting wild resources and breeding endangered plants.

## 2. Results

### 2.1. Ecological Factors

**Figure 1:**
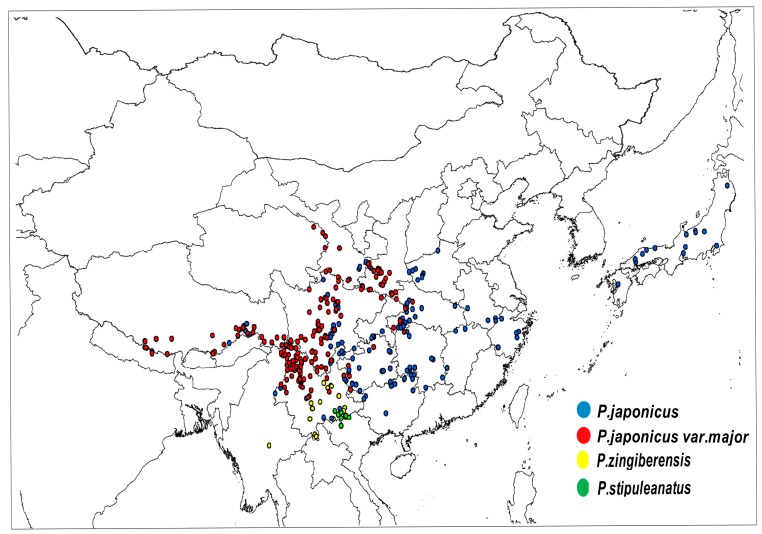
Global spatial distribution of sample points for four endangered *Panax* species, (blue) *P. japonicas*; (red) *P. japonicus var. major*; (yellow) *P. zingiberensis*; (green) *P. stipuleanatus*.

**Figure 2:**
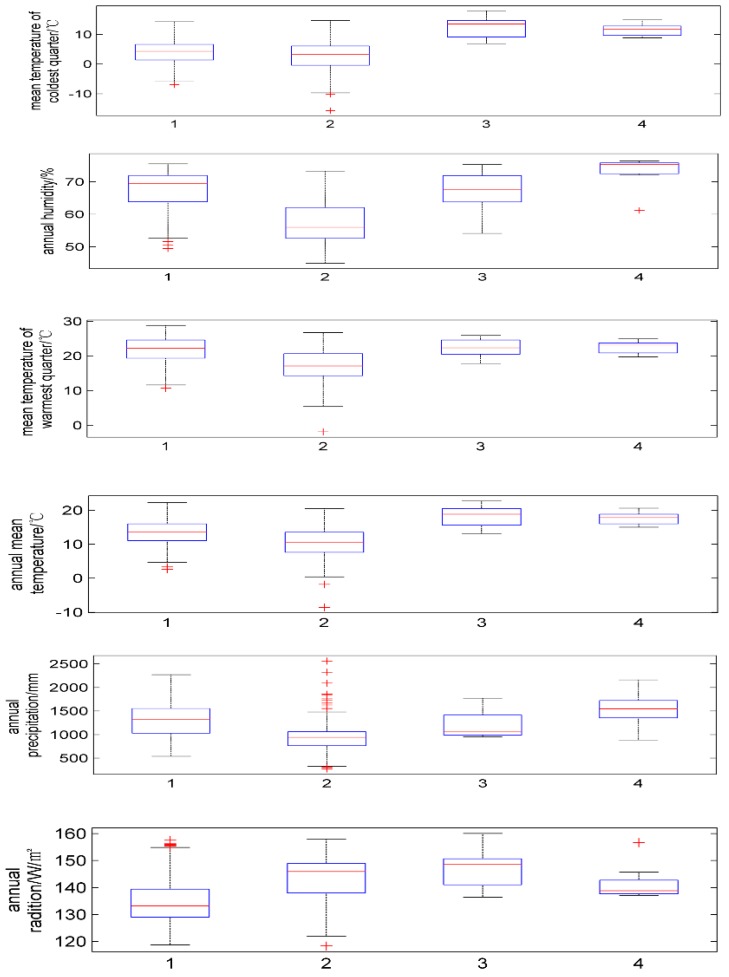
Boxplot of ecological factor of four endangered *Panax* species by MATLAB, ecological factor scope of *P. japonicus* and *P. japonicus* var. major are much broader(1: *P. japonicas*; 2: *P. japonicus var. major*; 3: *P. zingiberensis*; 4: *P. stipuleanatus*).

GMPGIS was used to extract the ecological factor data of collected sampling points (Figure 1 and Table 1), *Panax zingiberensis* and *Panax stipuleanatus* are distributed in low-latitude regions, which include China and Burma. *Panax japonicus* and *Panax japonicus var. major* are distributed in low and middle latitude regions. Figure 2 and Table 2 show the whole ecological factors range of each plant which plays an important role in cultivation. The contributions of each ecological factor were revealed by MaxEnt shown in Table 3, we found that the proportions of the mean temperature of the coldest quarter were 44.7%, 55.5%, 45.6%, and 43.0% for *P. japonicus* var. major, *P. japonicas*, *Panax zingiberensis*, *Panax stipuleanatus*. The proportions of annual precipitation were 36.0%, 37.8%, and 42.3% for *P. japonicas*, *Panax zingiberensis*, *Panax stipuleanatus*. This result provides a favorable condition for further analysis and experiment, and we can clearly understand the suitable range of climate factors and soil for these endangered medicinal plants.

### 2.2. Potential Distribution

#### 2.2.1. Global Potential Distribution

The potential distribution of endangered plants was influenced by the variation in the soil and climatic factors. Some environmental variables were also strongly correlated with the potential distributions.

**Figure 3:**
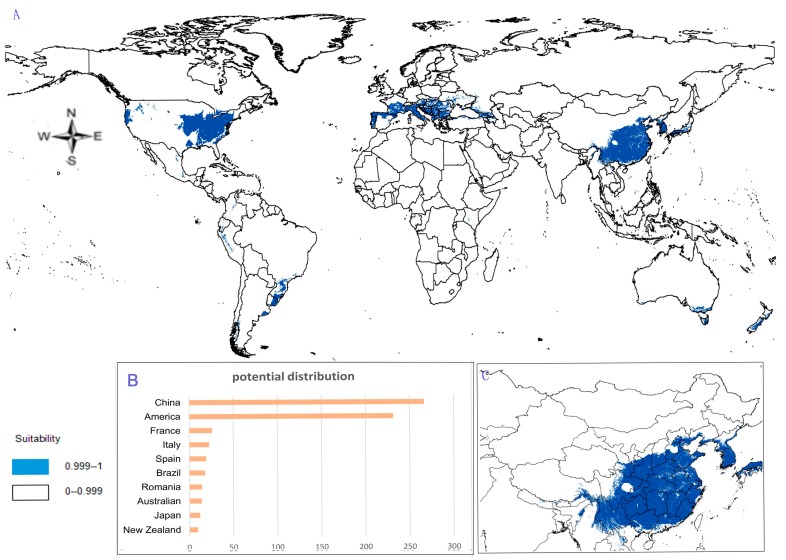
Spatial distribution map of potential suitable distribution for *P. japonicus* by GMPGIS. (**A**) Global potential distribution; (**B**) Global rank of the potential distribution area; (**C**) Chinese potential distribution.

**Figure 4:**
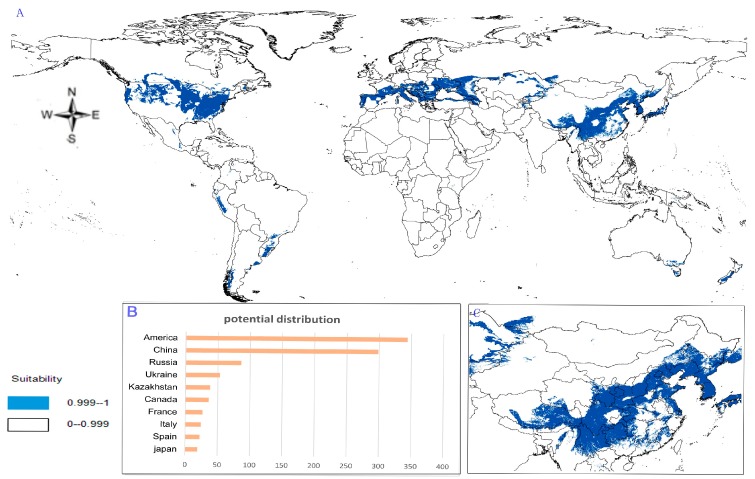
Spatial distribution map of potential suitable distribution for *P. japonicus var. major* by GMPGIS. (**A**) Global potential distribution; (**B**) Global rank of the potential distribution area; (**C**) Chinese potential distribution.

**Figure 5:**
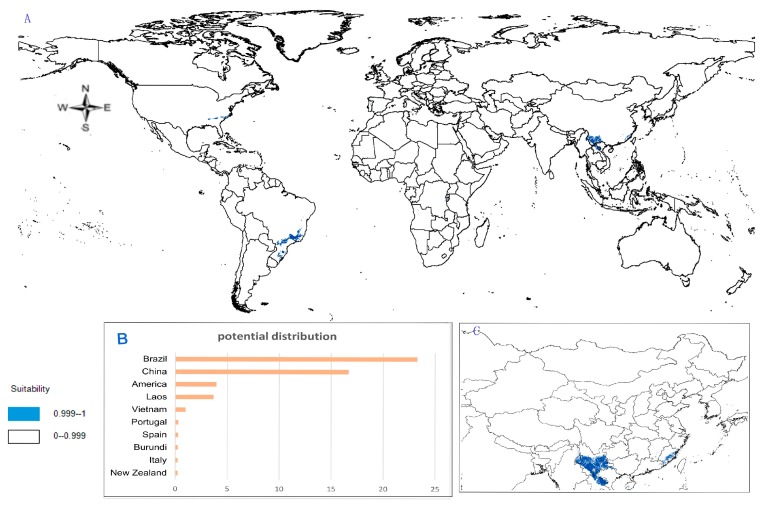
Spatial distribution map of potential suitable distribution for *P. zingiberensis* by GMPGIS. (**A**) Global potential distribution; (**B**) Global rank of the potential distribution area; (**C**) Chinese potential distribution.

**Figure 6:**
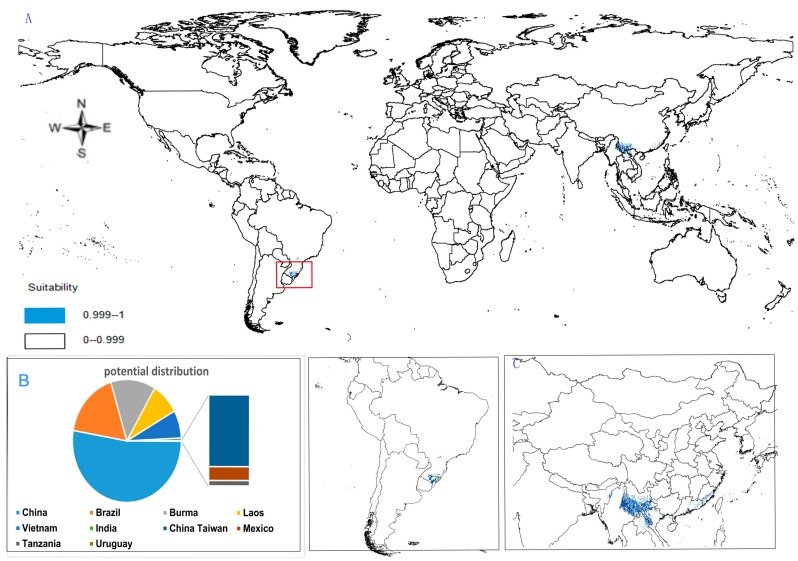
Spatial distribution map of potential suitable distribution for *P. stipuleanatus* by GMPGIS. (**A**) Global potential distribution; (**B**) Global rank of the potential distribution area; (**C**) Chinese potential distribution.

(1) Potential distribution of *P. japonicus*: The global potential distribution of *P. japonicus* is obtained by GMPGIS based on the ecological factors and soil, the total area is 118.29 × 10^5^ km^2^. *P. japonicus* is distributed mainly in North and South America, Asia, Europe, Oceania, and other regions (Figure 3A). The leading distribution areas are Southeast Asia and North America, which include China, Japan, South Korea, North Korea, the United States, and Canada. The top three distribution areas are China (2662.98 × 10^3^ km^2^), United States (2312.34 × 10^3^ km^2^), and France (260.81 × 10^3^ km^2^) as shown in Figure 3B.
(2) Potential distribution of *P. japonicus var. major*: The potential distribution regions for this plant are found in North America, Asia, and Europe (Figure 4A). The total global potential distribution area is 77.5 × 10^5^ km^2^, and the top three distribution areas are the United States (3438.73 × 10^3^ km^2^), China (2986.11 × 10^3^ km^2^), and Russia (861.09 × 10^3^ km^2^) (Figure 4B).
(3) Potential distribution of *P. zingiberensis*: The global potential suitable distribution of *P. zingiberensis* is obtained by GMPGIS (Figure 5A). The map shows the limited distribution of this species, which is distributed in a small part of Asia and South America. The total global potential suitable distribution area is 5.09 × 105 km^2^ ,the top three distribution areas are Brazil (232.79 × 10^3^ km^2^), China (166.71 × 10^3^ km^2^), and the United States (39.58 × 10^3^ km^2^) shown in Figure 5B. Thus, the introduction and cultivation of *P. zingiberensis* should be prioritized in these countries and regions.
(4) Potential distribution of *P. stipuleanatus*: The global potential suitable distribution of this plant is limited to several countries in Asia and South America (Figure 6A), the total area is 2.05 × 10^5^ km^2^. The top three distribution areas are China (108.03 × 10^3^ km^2^), Brazil (35.92 × 10^3^ km^2^), and Burma (27.33 × 10^3^ km^2^) (Figure 6B).

#### 2.2.2. Chinese Potential Distribution

*P. japonicus* and *P. japonicus var. major* are mainly distributed in the middle-latitude regions of China, including Sichuan Province, Guizhou Province, Shanxi Province, Shandong Province, Hebei Province, and Yunnan Province (Figure 3C and Figure 4C). However, the ecological adaptation areas of *P. zingiberensis* and *P. stipuleanatus* are limited and mainly distributed in Yunnan Province, Guangdong Province, and Fujian Province (Figure 5C and Figure 6C). In addition, *Panax japonicus C. A. Mey* is the most widely distributed species with ecological adaptation area of 2986.11 × 10^3^ km^2^ in China.

## 3. Discussion

### 3.1. Impact of Environmental Variables on Medicinal Plants

As shown in Table 1, different plants need different climates and habitats for their growth. The climatic factors of an actual area were simulated to provide a scientific basis for the cultivation of high-quality plants. The simulation was conducted to avoid blind introduction and reduced the workload. And we found that the mean temperature of the coldest quarter is the most important factor for these plants according to environment variable contributions (Table 3). Annual precipitation is also important for *P. zingiberensis* and *P. stipuleanatus*, *Panax japonicas*, annual average radiation is important for *Panax japonicus var. major*. Four endangered plants need suitable levels of humidity and rainfall. However, *P. stipuleanatus* requires a relatively low mean temperature of the coldest quarter, annual radiation, and high humidity, which are conditions found in damp habitats.

*Panax* species are ecologically fragile plants and sensitive to ecological factors. Due to excessive harvesting and lack of environmental protection, their natural distribution is limited. Previous studies on the relationship between plant quality and ecological factors indicate that temperature is the important ecological factor [29], similarities or differences in ecological factors lead to different metabolic contents and thus different effects. The metabolic contents of plants are affected by its habitat [30], and related research shows that lower temperature may lead to higher accumulation of ginsenosides in a certain scope of temperature for *Panax* species [31,32]. The present study shows that temperature is one of the important ecological factors, and the mean temperature of the coldest quarter is most important for these *Panax* species. The Light affects photosynthesis, changes the temperature, and affects the normal development of plants [33]. Rainfall influences humidity and other ecological factors [34]. The results of the previous study show a strong correlation between ecological factors and cultivation [35], in addition, all ecological factors interact with each other and are not isolated [36].

### 3.2. Distribution of Suitable Habitats

In this research, the suitable distribution areas of four plants were mapped based on the ecological factors. The suitable distribution areas of *P. japonicus* and *P. japonicus var. major* are mainly located in subtropical monsoon climate areas in China, followed by tropical monsoon climate zones, such as Sichuan Province, Guizhou Province, Shanxi Province, Shandong Province, Hebei Province, and Yunnan Province (Figure 3C and Figure 4C). However, some research shows that *P. japonicus var. major* distributed in the eastern Himalayas to western mountains in China, *P. japonicus* is the most widely distributed plant in China [37]. The suitable distribution of this study covers the above areas, and its potential suitability areas are mainly located north of Yangtze River, which mainly runs southwest to northeast across the province. Hence, the introduction and cultivation of *P. japonicus var. major* and *P. japonicus* should be prioritized in the above areas to obtain high output and solve the grim problems of resources. However, *P. zingiberensis* and *P. stipuleanatus* have limited potential suitable areas and are mainly distributed in Yunnan Province, Guangdong Province, and Fujian Province. Perhaps the *Panax* species in middle and low latitude can’t fully grow in cold areas, *Panax* species in high latitude are more resistant to cold.

**Figure 7:**
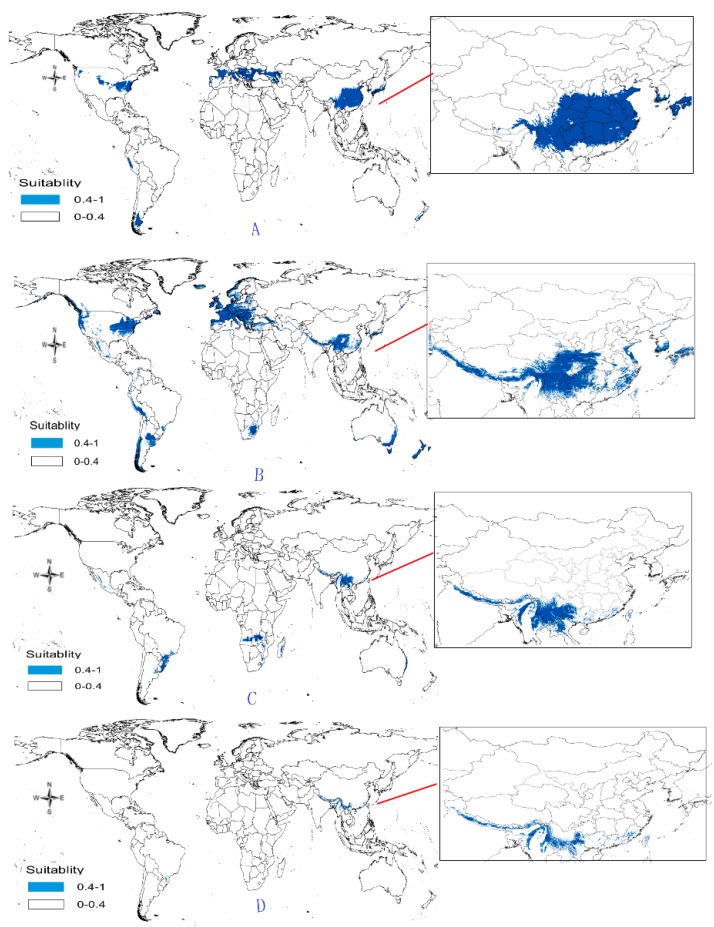
Spatial distribution map of potential distribution for four endangered *Panax* species based on MaxEnt. (**A**: *P. japonicas*; **B**: *P. japonicus var. major*; **C**: *P. zingiberensis*; **D**: *P. stipuleanatus*).

According to the ecological adaptability map based on GMPGIS, the introduction and cultivation of *P. japonicus* and *P. japonicus var. major* may also be feasible in The United States, Italy, Spain, Brazil, and Japan. The global potential distributions are 118.29 × 10^5^ and 77.5 × 10^5^ km^2^ for *P. japonicus var. major* and *P. japonicus*, respectively. *P. zingiberensis* and *P. stipuleanatus* exist with global potential distributions of 5.09 × 10^5^ and 2.05 × 10^5^ km^2^, respectively, and may be cultivated in Brazil and Laos. This study found many undiscovered potential areas, which provide favorable conditions for the introduction of these species and play an important role in strengthening the links between China and other countries. There are some small differences, the general trend of suitable distribution by GMPGIS is similar to MaxEnt’s (Figure 7), and these *Panax* species mainly distributed in middle and low latitudes regions. The suitable distribution only represents areas with similar environmental conditions in the sample area, but does not consider the factors of the genetic variation. Therefore, the predicted results may be deviated from the actual adaptation area of the plant. It needs to be studied in depth or be cultivated in the potential distribution to identify suitable areas for introduction and cultivation. Considerable research was conducted on *Panax* species for its development and utilization because its resources have received attention worldwide. With regard to unexploited wild plants, protective measures should be taken, and environmental protection should be strengthened.

### 3.3. Features of GMPGIS

We used GMPGIS to study the ecological suitability of *P. ginseng*, *P. quinquefolium*, *Radix Astragali* and effectively guide the production layout of the medicinal herbs [38,39]. The GMPGIS has the following festures:

(1) GMPGIS adopts the multi-index comprehensive evaluation for the quantitative and spatial analyses of the four medicinal plants.
(2) The results intuitively show the range of ecological factors and the best potential ecological areas of the plants.
(3) GMPGIS also explores the area with similar climates and soils of sampling points for medicinal plants.
(4) This system houses over 240 medicinal plants global sampling points.
(5) The model has high accuracy for medicinal plants.

The system includes ecological and environmental databases that facilitate the cultivation of medicinal plants. With the constant update and improvement of global climate database and soil data, GMPGIS technology will play an important role in the production of Chinese herbal medicine. We can intuitively understand the differences and used them to predict the distributions of medicinal plants.

## 4. Methods

**Figure 8:**
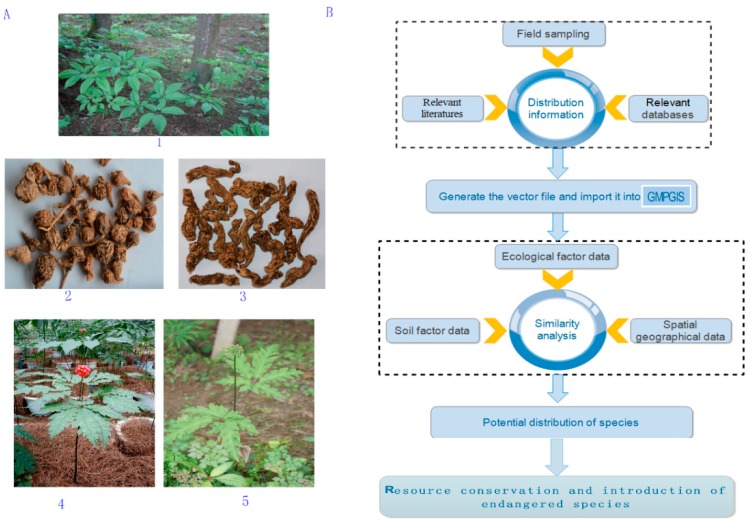
Picture of plant and materials (**A**) (1: *P. japonicus*, 2, 5: *P. japonicus var. major*, 3: *P. zingiberensis*, 4: *P. stipuleanatus*), and flow-chart of potential distribution similarity analysis (**B**).

Distribution sites of plants were collected from relevant literature and databases. Subsequently, their similarities were calculated after importing the latitude and longitude values from the ecological database into the GMPGIS. Finally, the global potential distribution and ecological factor values of the four endangered plants were obtained (Figure 8). The results were verified by MaxEnt.

### 4.1. GMPGIS Database

The ecological databases are important for the ecological suitability analysis. Climate data were mainly obtained from global climate data (WorldClim) and global climatologies for bioclimatic modeling (CliMond). WorldClim is recognized by the international community as a regional and accurate climate database and was established by Robert J. Hijmans [40], Susan Cameron, and Juan Parra of the Museum of Vertebrate Zoology (MVZ) , University of California, Berkeley, United States. CliMond aims to share different formats of environmental data, modeling tools, and meteorological expertise for ecological studies, such as species distribution models, species endangerment models, and global climate change. This database provides data such as the monthly minimum temperature, monthly maximum temperature, monthly precipitation, monthly average, 9 a.m. relative humidity, monthly average, 3 p.m. relative humidity, and 35 bioclimatic data layers.

Soil data were mainly obtained from the Harmonized World Soil Database, which is jointly formed by the United Nations Educational, Scientific, and Cultural Organization and the International Institute for Applied Systems Analysis. These institutions provide data, such as soil name, texture, effective water content, organic matter, pH, conductivity, and other indicators. The soil types include Andosols, Acrisols, Alisols, Arenosols, Anthrosols, Chernozems, Calcisols, Cambisols, Fluvisols, Greyzems, Ferralsols, Regosols, Solonchaks, Solonetz, Gleysols, Gypsisols, Histosols, Leptosols, Kastanozems, Luvisols, Lixisols, Phaeozems, Planosols, Plinthosols, Nitisols, Podzoluvisols, Podzols, and Vertisols.

The model selected seven ecological factors, including the mean temperature of the coldest quarter, annual mean temperature, annual precipitation, annual average radiation, annual humidity, and soil type for the analysis of these plants.

### 4.2. Data Collection

Data on the distributions of *P. japonicus, P. japonicus var. major, P. zingiberensis*, and *P. stipuleanatus* were collected from our field sampling results combined with the data from the Chinese Virtual Herbarium (CVH), National Specimen Information Infrastructure (NSII), and Global Biodiversity Information Facility (GBIF). A total of 176 sampling points for *P. japonicus*, 274 points for *P. japonicus var. major*, 102 points for P. zingiberensis, and 101 points for P. stipuleanatus were collected and analyzed for the global ecological suitability.

### 4.3. Principle and Algorithm of GMPGIS

Cluster analysis is the process of partitioning a set of data points (or observations) into subsets, the k-means method is the most famous among clustering algorithms. For the original algorithm, formally, a centroid-based partitioning technique uses the centroid of a cluster *C_i_* to represent that cluster, where *E* is the sum of the squared error for all points in the data set; *p* is the point in space representing a given object; and conceptually, the centroid of a cluster is its center point defined as follows:

(1) $$E=\overset{k}{\underset{i=1}{\sum }}\underset{p\in C_{i}}{\sum }dist{\left(p,c_{i}\right)}^{2}$$

The GMPGIS aim to predict the distribution of medicinal plants. Firstly, to eliminate the influence of a different method, the processing of ecological factors must be standardized. Secondly, to improve the k-means, a range-based technique is adopted to evaluate the ecological suitability models and to make them more adaptable to potential distribution prediction. Thirdly, the clustering layer is reclassified, and the potential distribution of areas is discovered. Fourthly, the suitable soil layer are intersected with the climatic factors in a Euclidean distance layer. The main steps of the model by ArcGis 10.2 are as follows:

(1) Step 1: Linear normalization is performed on the original data. Suppose that *min_A_* and *max_A_* were the minimum and maximum values of a layer A. Linear normalization maps a value *V_i_* of A to *V_i_* in the range [*newmin_A_, newmax_A_*] by computing the following:
  (2) $$V_{i}^{\prime }=\frac{V_{i}^{\prime }-min_{A}}{max_{A}-min_{A}}\times 100$$
(2) In our study, an improved k-means was adopted to evaluate the ecological suitability models. A range-based partitioning technique uses the critical size of a cluster *Di* to represent that cluster. Conceptually, the critical of a cluster is its marginal value *di*. The range can be defined in various ways, such as by the polyhedron assigned to the cluster. The difference between an object *p* ∊ *Di* and *di,* the representative of the cluster, is measured by dist (*p*, *di*), where dist (*x, y*) is the Euclidean distance between two points, *x* and *y*. The quality of cluster *Di* can be measured by the within-cluster variation, which is the sum of the squared error between all points in *Di* and the range *di*, defined as follows:
  (3) $$E=\overset{k}{\underset{i=1}{\sum }}\underset{p\in D_{i}}{\sum }dist{\left(p,d_{i}\right)}^{2}$$
  where
  (4) $$dist\left(p,d_{i}\right)=\mathrm{IF}\left[min\leq v_{i}\leq max,0,\min(\left|v_{i}^{\prime }-newmin_{A}\right|,\left|v_{i}^{\prime }-newmin_{A}\right|\right]$$
  where *E* is the sum of the *squared error* for all points in the data set, *p* is the point in space representing a given object, and *di* is the range of cluster *Di* (both *p* and *di* were multidimensional). *newmin_A_* is the minimum value after standardizing the layer; *newmax_A_* is the maximum value after standardizing the layer.
(3) According to the results of the distance calculation [*Min_d_*, *Max_d_*], the grid was classified, and the most similar ecological area was discovered.
(4) The suitable soil layer and climatic factors in the Euclidean distance layer were intersected.

### 4.4. Analysis by MaxEnt

The data of sampling points and environment variables were imported into the MaxEnt software. Parameter settings were as follows: the training set is 75% of the sampling point data, the test set is the remaining 25% used to examine the predictive ability of the model, and the jackknife was used to test the weight. The threshold-independent receiver operating characteristic and area under the receiver operating characteristic curve values were calculated by MaxEnt. The results were analyzed by ArcGIS 10.2, and the maps of ecologically suitable regions for endangered plants were obtained based on the main ecological factors.

## 5. Conclusions

The growth of endangered medicinal plants requires appropriate ecological conditions. Their quality are closely related to ecological factors such as climate and soil. The suitability distribution analysis of medicinal plants has been based on the traditional experience, single ecological factor, and single origin studies. Therefore, traditional analysis shows low efficiency and poor accuracy. GMPGIS can combine the attributed and graphic data with powerful spatial data management, analysis and mapping ability. The prediction of potential suitable distributions is conducive to the conservation of biological diversity. The results of the present analysis include output maps, charts, and other forms, which reduce the statistical and mapping work, and achieve the visualization of regionalization for these *Panax* species. Hidden information can be revealed by the suitability analysis of producing areas from the geographic information system for Chinese traditional medicines. This study greatly improves the management level and application value of the resource information for traditional Chinese medicines, and provides a reference for resource conservation, introduction, and cultivation of endangered *Panax* species. This study is just for protection of the species, and the scientific research, it is not open to other harmful behavior.

## Figures and Tables

**Table 1:** Global spatial distribution of sample points for four endangered *Panax* species.

Latin Name of Species	Sampling Distribution	Sampling Points
*P. japonicus*	Global: China, Japan, North Korea China: Yunnan, Guizhou, Sichuan, Hubei, etc.	176
*P. japonicus var.major*	China: Shaanxi, Sichuan, Yunan, Gansu, Guizhou, etc.	221
*P. zingiberensis*	Global: China, Burma China: Yunnan	102
*P. stipuleanatus*	Global: China, Burma China: Yunnan	101

**Table 2:** The range of ecological factors for four endangered *Panax* species.

Latin Name of Species	Annual Mean Temperature/°C	Mean Temperature of Coldest Quarter/°C	Mean Temperature of Warmest Quarter/°C	Annual Precipitation/mm	Annual Humidity/%	Annual Average Radiation/w·m^−2^
*P. japonicus*	2.6~22.3	−7.0~14.3	10.7~28.8	539~2273	49.4~75.5	118.7~157.6
	soil types: Lixisols, Arenosols, Chernozems, Luvisols, Ferralsols, Acrisols, Andosols, etc.					
*P. japonicus var.major*	−8.6~20.5	−17.5~14.7	−1.9~26.7	272~2562	44.9~73.3	118.3~157.9
	soil types: Lixisols, Chernozems, Greyzems, Leptosols, Arenosols, etc.					
*P. zingiberensis*	13~22.7	6.9~18.0	17.8~25.9	957~1772	54.1~75.3	136.3~160.1
	soil types: Acrisols, Arenosols, Arenosols, etc.					
*P. stipuleanatus*	15~20.6	8.8–14.9	19.7~24.9	888~2161	61.2~76.4	136.9~156.0
	soil types: Acrisols, Arenosols, etc.					

**Table 3:** Contribution of ecological factors (%) based on MaxEnt.

Variable	Contribution (%)			
	*P. japonicus*	*P. japonicus var. major*	*P. zingiberensis*	*P. stipuleanatus*
Cold	55.5	44.7	45.6	43.0
Pre	36.0	5.7	37.8	42.3
Tem	4.5	7.1	4.9	2.8
Warm	3.0	4.5	9.4	9.7
Rad	0.6	37.7	2.0	0.1
hum	0.4	2.4	0.3	2.0

(Cold: mean temperature of coldest quarter, pre: annual precipitation, tem: annual mean temperature, warm: mean temperature of warmest quarter, rad: annual average radiation, hum: annual humidity).

## Supplementary Materials

Supplementary File 1
