# Supplementary material for: Predicting the Global Potential Distribution of Four Endangered Panax Species in Middle-and Low-Latitude Regions of China by the Geographic Information System for Global Medicinal Plants (GMPGIS)
Source: Molecules. 2017 Sep 28;22(10):1630. doi: 10.3390/molecules22101630 (PMC6151741; doi:10.3390/molecules22101630)
Supplement: Supplementary File 1 [file molecules-22-01630-s001.zip › responses to reviewers’commens/Firet response to revises/molecules-212648.docx]

Article

Predicting the global potential suitable distribution of four endangered *Panax* species in middle-and low-latitude regions of China by the geographic information system for global medicinal plants Firstname Zhixia Du^1^, Jie Wu^2^, Xiangxiao Meng^2^, Jinhua Li^1^ and Linfang Huang ^1^*

1 Institute of Medicinal Plant Development, Chinese Academy of Medical Sciences (CAMS), Peking Union Medical College (PUMC); zxdu712@126.com.

2 Institute of Chinese Materia Medica, China Academy of Chinese Medical Sciences, Beijing, China；jwu1986@icmm.ac.cn.

2 Institute of Chinese Materia Medica, China Academy of Chinese Medical Sciences, Beijing, China; [xxmeng@icmm.ac.cn](mailto:xxmeng@icmm.ac.cn).

1 Institute of Medicinal Plant Development, Chinese Academy of Medical Sciences (CAMS), Peking Union Medical College (PUMC); jinhuali108@126.com.

*Correspondence: lfhuang@implad.ac.cn; Tel.: +86-10-57833197

Academic Editor: name

Received: date; Accepted: date; Published: date

**Abstract:** Global biodiversity is strongly influenced by the decrease in endangered biological species. Predicting the distribution of endangered medicinal plants is necessary for resource conservation. A spatial distribution model, geographic information system for global medicinal plants (GMPGIS), was used to predict the global potential suitable distribution of four endangered *Panax* species, including *Panax japonicus (T. Nees) C. A. Meyer* and *Panax japonicus var. major (Burkill) C. Y. Wu & K. M. Feng* distributed in low-and middle -latitude and *Panax zingiberensis C. Y. Wu & K. M. Feng* and *Panax stipuleanatus C. T. Tsai & K. M. Feng* in low-latitude regions of China using7 bioclimatic variables and 600 occurrence points. Results indicate the limited distribution of these plants. The areas are 118.29 × 10^5^ km^2^ and 77.5 × 10^5^ km^2^ for *P. japonicus* and *P. japonicus* var. major, respectively, which are mainly distributed in the United States, China, and Japan. By contrast, the areas for *P. zingiberensis* and *P. stipuleanatus* are 5.09 × 10^5^ km^2^ and 2.05 × 10^5^km^2^, respectively, which are mainly distributed in China and Burma. *P. japonicus* has the widest distribution among the four species. This study suggests the potential suitable distribution area as the suitable cultivation area for these endangered plants to solve the resource problem. The mean temperature of coldest quarter and the annual precipitation are found to be the most critical factors. This scientific prediction can be used as reference for resource conservation of endangered plants and as a guide to search for endangered species in previously unknown areas.

**Keywords:** *Panax*, global potential distribution, geographic information system for global medicinal plants

1. Introduction

The resources of *panax (Araliaceae)* are the important medicinal resources in the world. Species such as *Panax* ginseng and *Panax quinquefolium* are distributed at high-latitude regions, whereas *Panax japonicus (T. Nees) C. A. Meyer*, *Panax japonicus var. major (Burkill) C. Y. Wu & K. M. Feng, Panax zingiberensis C. Y. Wu & K. M. Feng* and *Panax stipuleanatus C. T. Tsai & K. M. Feng* are found in middle- and low-latitude regions. Some endangered *Panax* species, such as *P. japonicus* and *P.* japonicus var. major found in middle- and low-latitude regions, were included in the Chinese Pharmacopoeia. *P. japonicus* is known as “the king of herbs” in Chinese folk, is widely used as a traditional Japanese medicine, and is included in the Japanese Pharmacopoeia. *P.* *zingiberensis* and *P. stipuleanatus* are also widely used as traditional ethnic medicines. Panax species[[1-4](#_ENREF_1)], including *P. ginseng, P. quinquefolius, P. japonicus, P. japonicus var. major, and P. stipuleanatus*, are popular due to their potential medicinal properties, such as anti-fatigue, anti-tumor, anti-thrombotic, anti-inflammatory, anti-oxidative, and immune-enhancing effects and thus have a substantial market demand worldwide[[5-7](#_ENREF_5)]. However, their resources are recently declining due to excessive harvesting and lack of environmental protection. Therefore, predicting the distribution of these plants is necessary for conservation[[8](#_ENREF_8)].

With the development of network technology, distribution prediction model has become one of the common methods in biodiversity conservation, such as MaxEnt (Maximum Entropy), Random Forset [9-13], GMPGIS (the geographic information system for global medicinal plants) [[14-1](#_ENREF_9)6]. In this research, the global potential distribution of four endangered *Panax* species found in middle- and low-latitude regions was predicted by using GMPGIS. The GMPGIS model was created by the Institute of Chinese Materia Medica to predict the distribution of medicinal plants[[1](#_ENREF_12)7], the environment database of model mainly includes WorldClim, CliMond, HWSD. This model has been used for introduction and conservation of *Panax ginseng C. A. Mey* and *Panax notoginseng (Burk.) F. H. Chen*[[1](#_ENREF_13)8].By combining the climate and soil factors of sample points to explore area that have the most similar ecological factors，we can determine the suitable environment for medicinal plants to scientifically and effectively protect and cultivate the endangered plants. MaxEnt is also widely used to predict species distribution [[19-2](#_ENREF_14)8]. Potential suitable distribution plays an important role in resource protection and cultivation of endangered plants. We studied and compared the differences between GMPGIS and MaxEnt and found that GMPGIS shows higher precision than MaxEnt. In this research, GMPGIS was used to analyze the plant habitats, whereas MaxEnt was used for supplementary information.

We used GMPGIS to scientifically predict the global distribution of four endangered Panax species based on the climate and soil factors. The global potential suitable distributions of the four plants were mapped by excluding the unsuitable areas, such as lakes, rivers, and cities. This research aims to predict the global potential suitable distribution of four endangered Panax species and provide a scientific reference for protecting wild resources and for breeding endangered plants.

2. Results

*2.1 Ecological factors*

GMPGIS was used to extract the ecological factor data from collected sampling points (figure1) to obtain the range of ecological factors (table1and figure 2).*Panax zingiberensis* and *Panax stipuleanatus* distributed in low-latitude regions, include china and Burma. *Panax japonicus* and *Panax japonicus* var. major distributed in low and middle latitude (figure 1). The contributions of each ecological factor were revealed by MaxEnt， we found that the proportions of the mean temperature of the coldest quarter and annual average radiation were 44.7%, 37.7%for *P. japonicus* var. major, the proportions of the mean temperature of the coldest quarter and annual precipitation were 55.5%, 36.0%for *P. japonicas*, the proportions of the mean temperature of the coldest quarter and annual precipitation were 45.6%,37.8%for *Panax zingiberensis*, the proportions of the mean temperature of the coldest quarter and annual precipitation were 43.0%,42.3%for *Panax stipuleanatus*(table2). Based on GMPGIS results, we can clearly understand the suitable range of climate factors and soil for these endangered medicinal plants (table1). This result provides a favorable condition for further analysis and experiment.


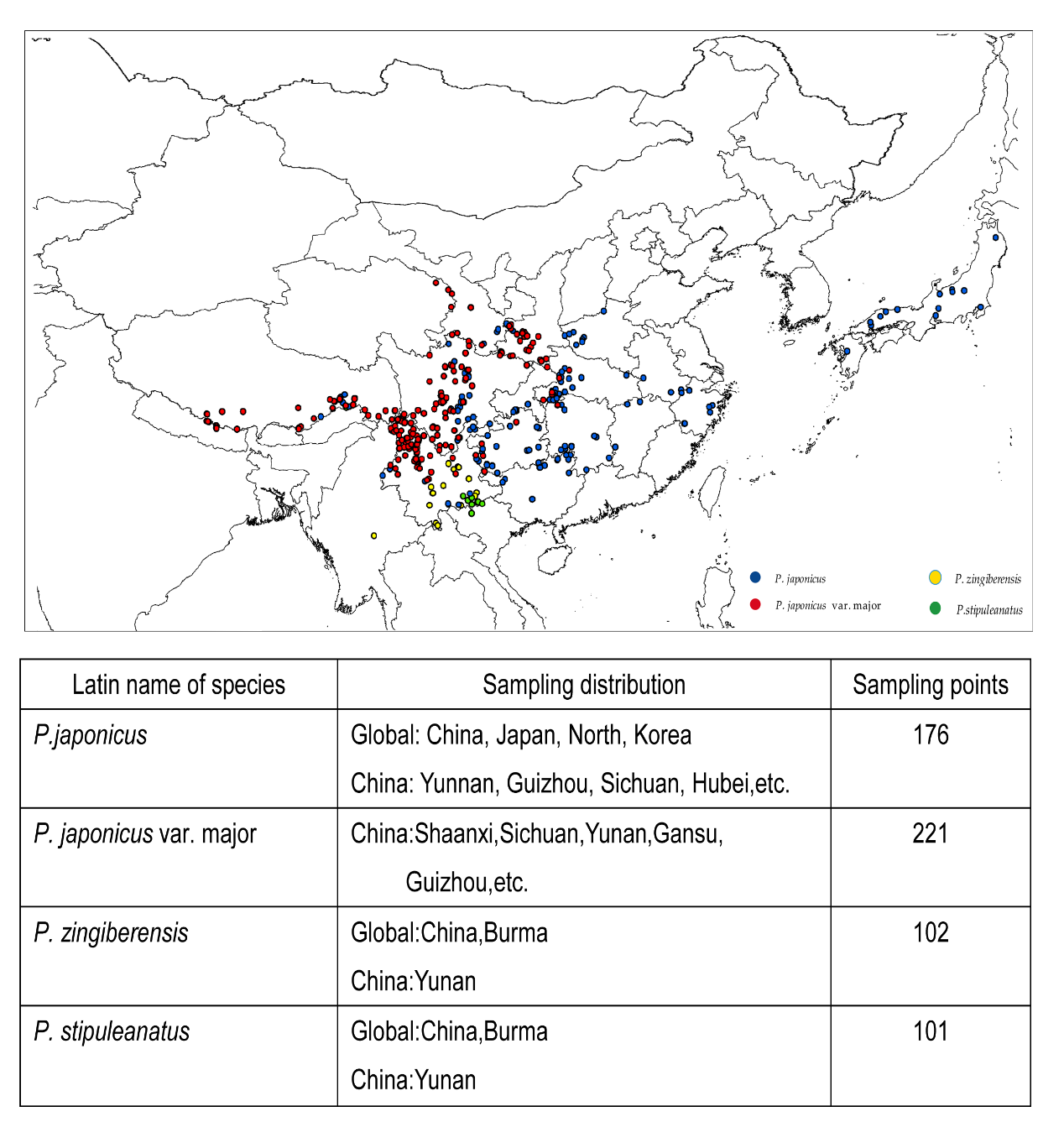


**Figure1:** Global spatial distribution of sample points for four endangered panax species. (Blue: P.japonicus, red: P.japonicus var.major, yellow: P.zingiberensis, green: P.stipuleanatus)

**Table1:** The range of ecological factors for four endangered panax species.

| Latin name of species | Annual mean temperature/℃ | Mean temperature of coldest quarter/℃ | Mean temperature of warmest quarter/℃ | annual precipitation/mm | annual humidity/% | annual average radiation/ w**^.^**m^-2^ |
| --- | --- | --- | --- | --- | --- | --- |
| P.japonicus | 2.6 ~22.3 | -7.0 ~ 14.3 | 10.7 ~ 28.8 | 539 ~2273 | 49.4 ~ 75.5 | 118.7~ 157.6 |
|  | soil types :Lixisols，Arenosols，Chernozems，Luvisols，Ferralsols，Acrisols，Andosols，etc. | | | | | |
| P.japonicus var.major | -8.6~20.5 | -17.5~14.7 | -1.9~26.7 | 272~2562 | 44.9~73.3 | 118.3~157.9 |
|  | soil types: Lixisols, Chernozems，Greyzems，Leptosols，Arenosols,etc. | | | | | |
| P.zingiberensis | 13~22.7 | 6.9~18.0 | 17.8~25.9 | 957~1772 | 54.1~75.3 | 136.3~160.1 |
|  | Types: Acrisols, Arenosols，Arenosols,etc. | | | | | |
| P.stipuleanatus | 15~20.6 | 8.8-14.9 | 19.7~24.9 | 888~2161 | 61.2~76.4 | 136.9~156.0 |
|  | soil types: Acrisols, Arenosols,etc. | | | | | |


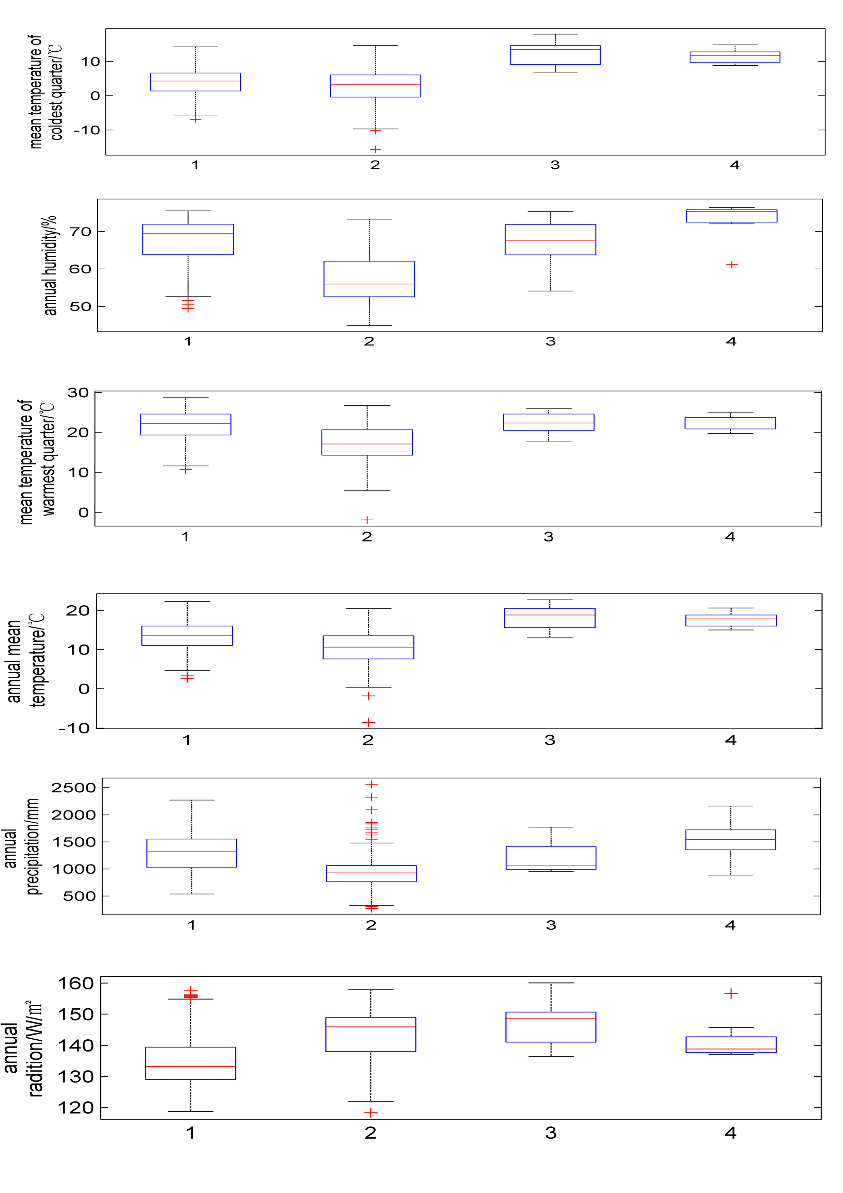


**Figure2:** Boxplot of ecological factor of four endangered panax species. (1: *P.japonicus*, 2: *P. japonicus var. major*, 3: *P. zingiberensis*, 4: *P. stipuleanatus*)

**Table2:** Contribution of ecological factors (%) based on MaxEnt.


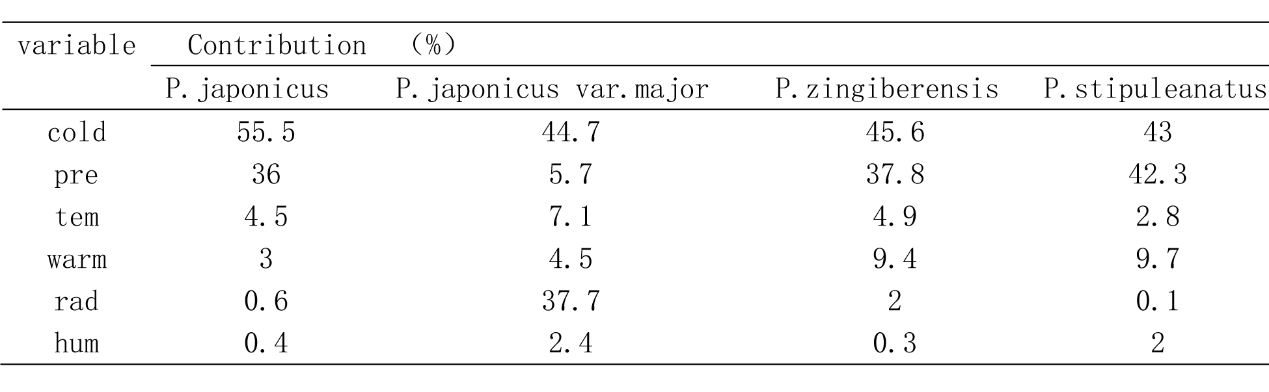


(Cold: mean temperature of coldest quarter, pre: annual precipitation, tem: annual mean temperature, warm: mean temperature of warmest quarter, rad: annual average radiation, hum: annual humidity)

2.2 Potential distribution

2.2.1 Global potential distribution

The potential distribution of the four endangered plants was influenced by the variation in the soil and climatic factors. Some environmental variables were also strongly correlated with the potential distributions.

(1) Potential distribution of *P. japonicus*: The global potential suitable distribution of *P. japonicus* was obtained by GMPGIS based on the range of ecological factors and soil, the total area is 118.29 × 10^5^ km^2^. *P. japonicus* was mainly distributed in North and South America, Asia, Europe, Oceania, and other regions (figure3A). The largest distribution area is found in Southeast Asia, including China, Japan, South Korea, and North Korea, and North America, including the United States and Canada. The top three distribution areas are China (2662.98 × 10^3^ km^2^), United States (2312.34 × 10^3^ km^2^), and France (260.81 × 10^3^ km^2^) (figure3B).


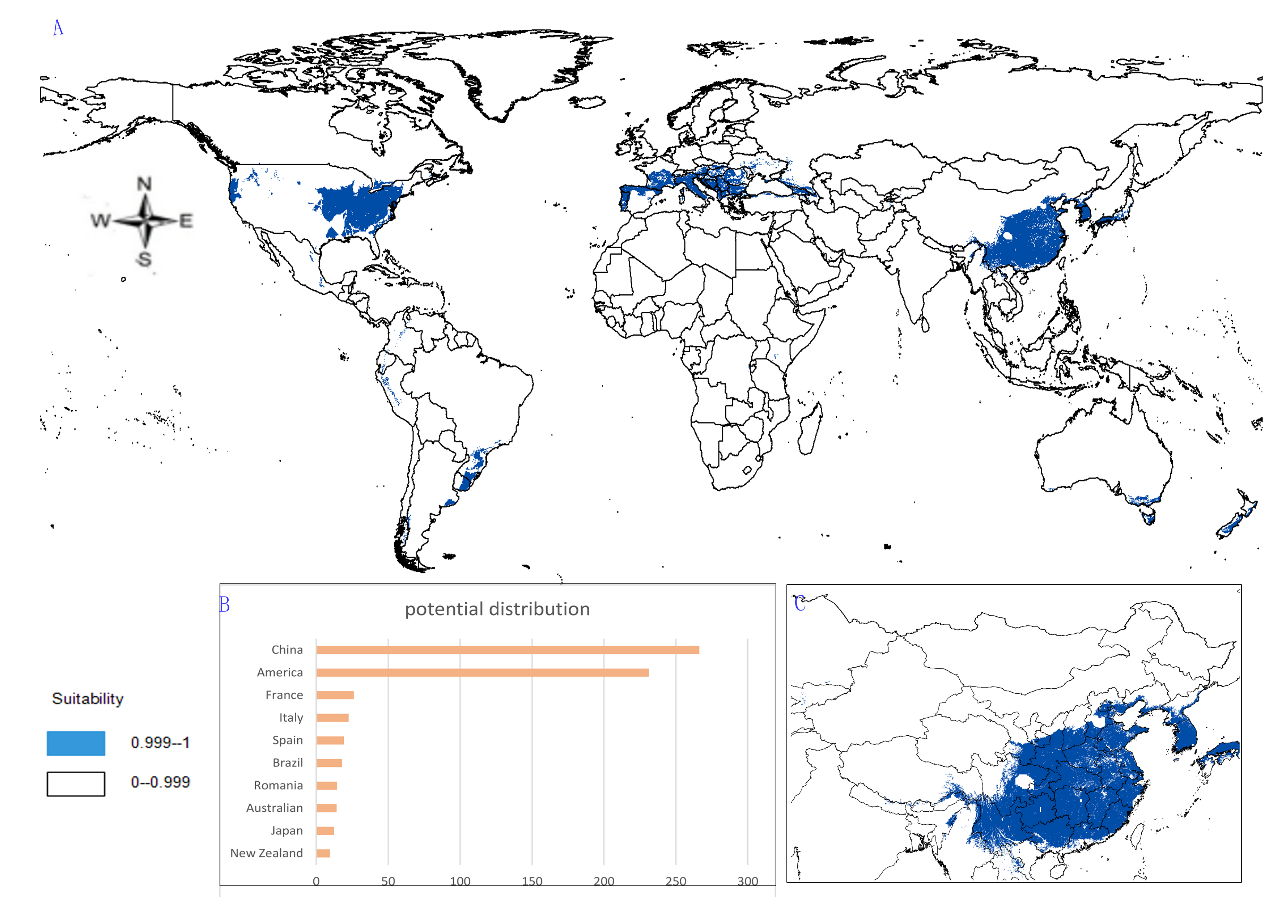


**Figure3:** Spatial distribution map of potential suitable distribution for *P.japonicus* by GMPGIS*. Global* potential distribution (A), Global rank of the potential distribution area (B), Chinese potential distribution(C).

(2) Potential distribution of *P. japonicus var. major*: The potential suitable distribution regions for this plant are North America, Asia, and Europe (figure4A). The total global potential suitable distribution area is 77.5 × 10^5^ km^2^ ,and he top three distribution areas are the United States (3438.73 × 10^3^ km^2^), China (2986.11 × 10^3^ km^2^), and Russia (861.09 × 10^3^ km^2^)(figure4B).


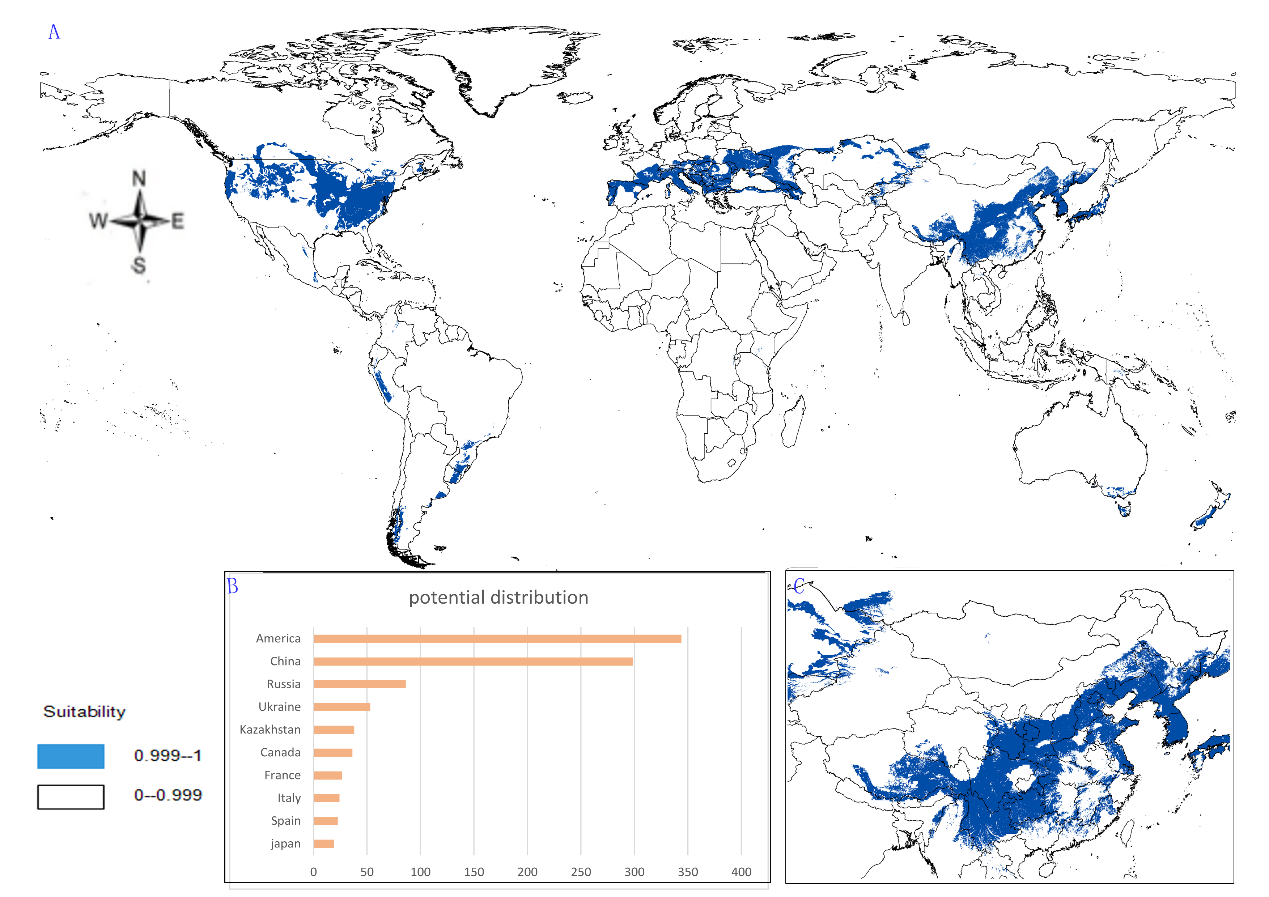


**Figure4:** Spatial distribution map of potential suitable distribution for *P. japonicus* var. major by GMPGIS*. Global* potential distribution (A), Global rank of the potential distribution area (B), Chinese potential distribution(C).


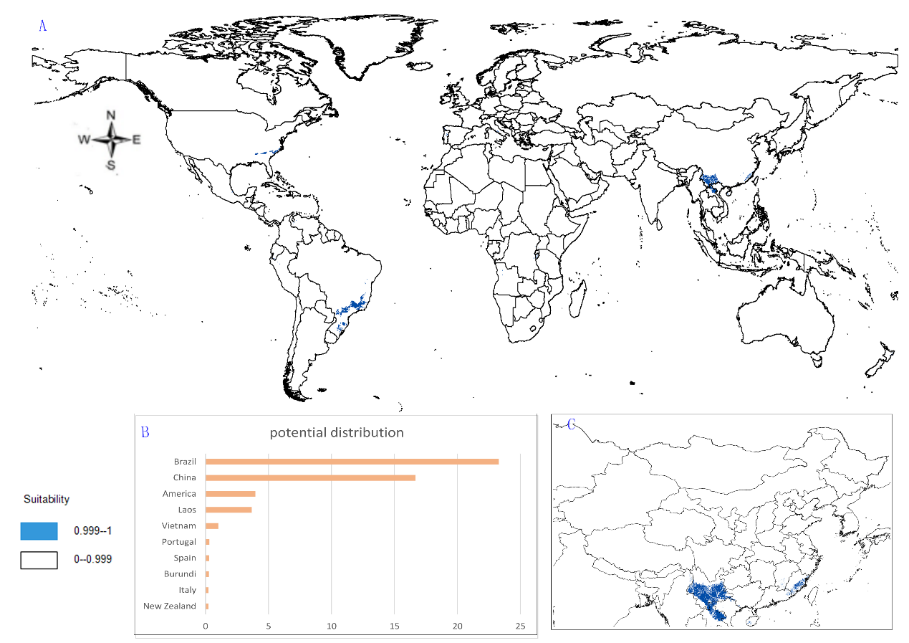


**Figure5:** Spatial distribution map of potential suitable distribution for *P. zingiberensis* by GMPGIS*. Global* potential distribution (A), Global rank of the potential distribution area (B), Chinese potential distribution(C).

(3) Potential distribution of *P. zingiberensis*: The global potential suitable distribution of *P. zingiberensis* was obtained by GMPGIS (figure5A). The map indicates the limited distribution of this species, which was distributed in a small part of Asia and South America. The total global potential suitable distribution area is 5.09 × 10^5^ km^2^ ,the top three distribution areas are Brazil (232.79 × 10^3^ km^2^), China (166.71 × 10^3^ km^2^), and United States (39.58 × 10^3^ km^2^)(figure5B). Thus, the introduction and cultivation of *P. zingiberensis* should be prioritized in these countries and regions.

(4) Potential distribution of *P. stipuleanatus*: The global potential suitable distribution of this plant is limited to several countries in Asia and South America (figure6A), the total area is 2.05 × 10^5^km^2^. The top three distribution areas are China (108.03 × 10^3^ km^2^), Brazil (35.92 × 10^3^ km^2^), and Burma (27.33 × 10^3^ km^2^) (figure6B).


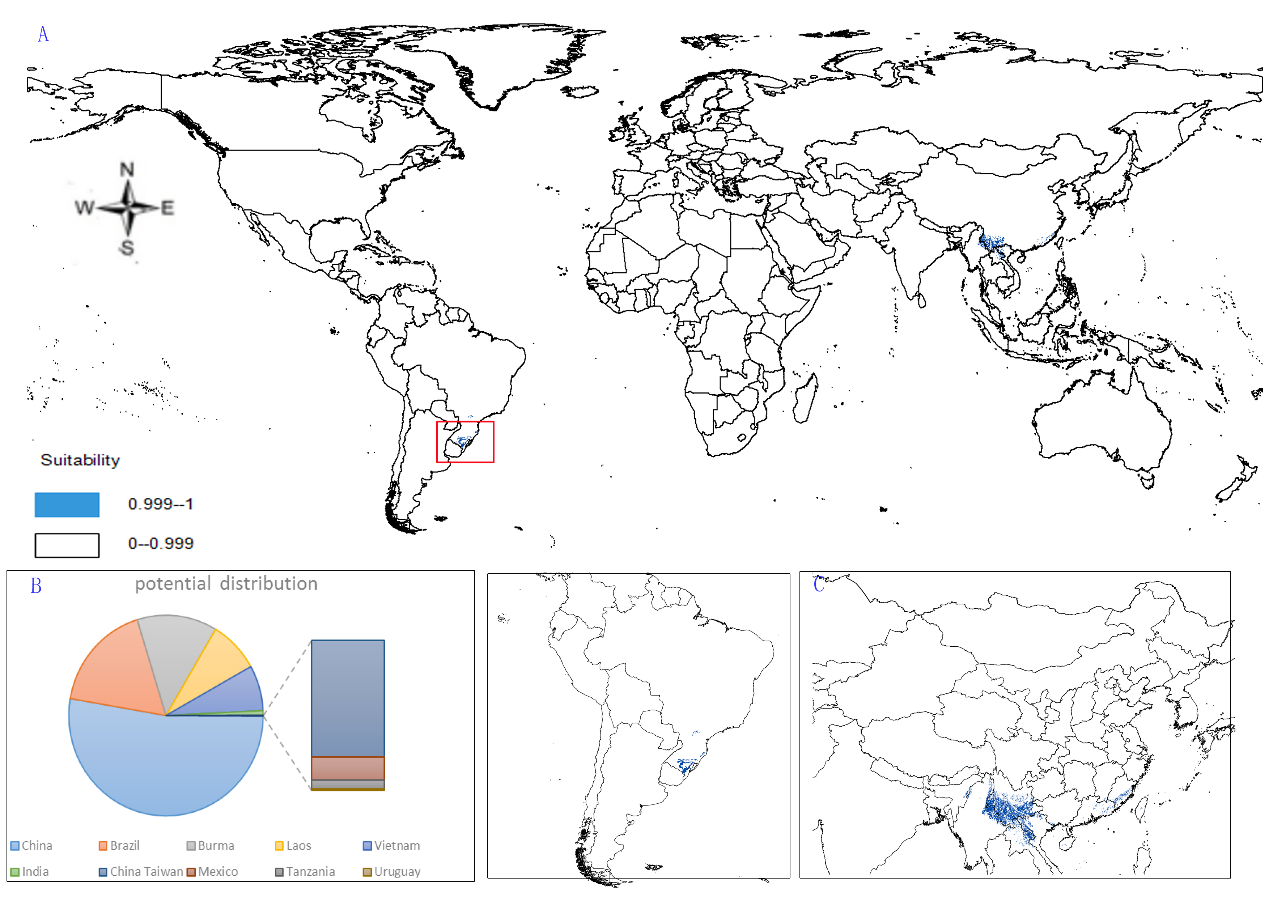


**Figure6:** Spatial distribution map of potential suitable distribution for *P. stipuleanatus* by GMPGIS*. Global* potential distribution (A), Global rank of the potential distribution area (B), Chinese potential distribution(C).

2.2.2 Chinese potential distribution

*P. japonicus* and *P. japonicus var. major* are mainly distributed in the middle-latitude regions of China, including Sichuan Province, Guizhou Province, Shanxi Province, Shandong Province, Hebei Province, and Yunnan Province (figure3C, figure4C). However, the ecological adaptation areas of *P. zingiberensis* and *P. stipuleanatus* are limited and are mainly distributed in Yunnan Province, Guangdong Province, and Fujian Province (figure5C, figure6C). In addition, *Panax japonicus C. A. Mey* is the most widely distributed species with ecological adaptation area of 2986.11 × 10^3^ km^2^ in China.

3. Discussion

3.1 Impact of environmental variables on medicinal plants

From the environment variable range for four plants (table1), we found that suitable environment for these plant growth, and different plants need different climates and habitats for their growth. The climatic factors of an actual area were simulated to provide a scientific basis for the cultivation of high-quality plants. From the environment variable contribution for four plants (table2), we found that the mean temperature of the coldest quarter is most important for these plants. However, annual precipitation also important for *P. zingiberensis* and *P. stipuleanatus*, *Panax japonicas*, annual average radiation is important for *Panax japonicus var. major* .The simulation was conducted to avoid blind introduction and reduce the workload. The four endangered plants need suitable levels of humidity and rainfall,however, *P. stipuleanatus* requires a relatively low mean temperature of coldest quarter, annual radiation, and high humidity, which are conditions found in damp habitats.

*Panax* species are ecologically fragile and sensitive to ecological factors, and due to excessive harvesting and lack of environmental protection, their natural distribution is limited. Previous studies on the relationship between plant quality and ecological factors indicate that temperature is the important ecological factor[[2](#_ENREF_24)9], similarities or differences in ecological factors lead to different metabolic contents and thus different effects. The metabolic contents of plants are affected by its habitat[30],related research shows that in a certain scope of temperature, lower temperature may lead to higher accumulation of ginsenosides for panax species[31,32]. The present study shows that temperature is one of the important ecological factors, and the mean temperature of the coldest quarter is most important for these panax species. Light affects photosynthesis, changes the temperature, and affects the normal development of plants[33], rainfall influences humidity and other ecological factors[34]. The results of the previous study show a strong correlation between ecological factors and cultivation[35], in addition, all ecological factors interact with each other and are not isolated[36].

3.2 Distribution of suitable habitats

In this research, the suitable distribution areas of four plants were mapped based on the ecological factors. The suitable distribution areas of *P. japonicus* and *P. japonicus var. major* in China are mainly located in subtropical monsoon climate areas, followed by tropical monsoon climate zones, such as Sichuan Province, Guizhou Province, Shanxi Province, Shandong Province, Hebei Province, and Yunnan Province (figure3 and figure 4). However, some research shows that *P. japonicus var. major* distributed in the eastern Himalayas to western mountains in China, *P. japonicus* is the most widely distributed plant in China[37], the suitable distribution of this study covers above areas, and its potential suitability areas are mainly located in the north of Yangtze River, which mainly runs along the southwest to northeast across the province. The introduction and cultivation of *P. japonicus var. major* and *P. japonicus* should be prioritized in the above areas to obtain high output and solve the grim problems of resources. However, *P. zingiberensis* and *P. stipuleanatus* have limited potential suitable areas and are mainly distributed in Yunnan Province, Guangdong Province, and Fujian Province. Perhaps the panax species in middle and low latitude can^,^t full grown in cold area, panax species in high latitude is more resistant to cold.

According to the ecological adaptability map based on GMPGIS, the introduction and cultivation of *P. japonicus* and *P. japonicus var. major* may also be feasible in the United States, Italy, Spain, Brazil, and Japan. The global potential distributions are 118.29 × 10^5^ km^2^ and 77.5 × 10^5^ km^2^ for *P. japonicus var. major* and *P. japonicus*, respectively. *P. zingiberensis* and *P. stipuleanatus* have global potential distributions of 5.09 × 10^5^ km^2^ and 2.05 × 10^5^ km^2^, respectively, and may be cultivated in Brazil and Laos. This study found many undiscovered potential areas, which provide favorable conditions for the introduction of these species and play an important role in strengthening the links between China and other countries. There are some small differences, the general trend of suitable distribution by GMPGIS is similar to MaxEnt^,^ s (figure7), these panax species mainly distributed in middle and low latitudes area. The prediction suitable distribution only represent areas with similar environmental conditions in the sample area, but not consider the factors of the genetic variation. Therefore, the predicted results may be deviated from the actual adaptation area of the plant. It need to study in depth or cultivated in the potential distribution to identify suitable area for introduction and cultivation. Considerable research was conducted on *Panax* species for its development and utilization because its resources have received attention worldwide. For wild plants that are not yet exploited, protective measures should be taken, and environmental protection should be strengthened.


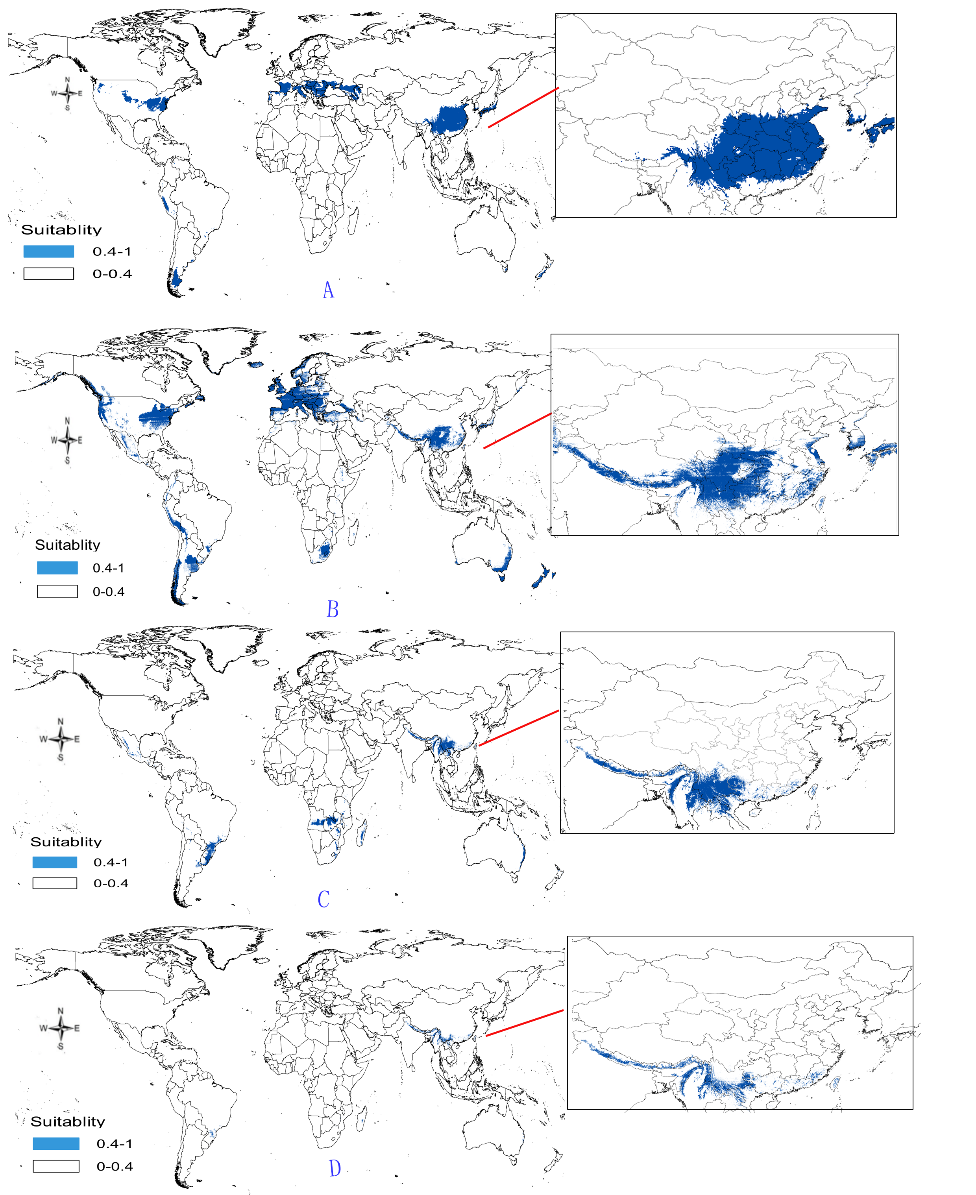


Figure7: Spatial distribution map of potential distribution for four endangered panax species based on MaxEnt. (A: *P.japonicus*, B: *P. japonicus var. major*, C: *P. zingiberensis*, D: *P. stipuleanatus*)

3.3 Features of GMPGIS

We used GMPGIS to study the ecological suitability of *P. ginseng*, *P. quinquefolium*, and *Radix Astragali* and effectively guide the production layout of the medicinal herbs[38,39].The GMPGIS has the following festures:

(1)GMPGIS adopts the multi-index comprehensive evaluation for the quantitative and spatial analyses of the four medicinal plants.

(2)The results intuitively show the range of ecological factors and the best potential ecological areas of the plants.

(3)GMPGIS also explores the area with similar climates and soils of sampling points for medicinal plants.

(4) This system houses over 240 medicinal plants global sampling points.

(5)The model has high accuracy for medicinal.

The system includes ecological and environmental databases that facilitate the cultivation of medicinal plants. With the constant update and improvement of global climate database and soil data, GMPGIS technology will play an important role in the production of Chinese herbal medicine. We can intuitively understand the differences and used them to predict the distributions of medicinal plants.

**4. Methods**

The ecological suitability of the four endangered plants was analyzed, and their distribution sites were collected from relevant literature and databases. Subsequently, their similarities were calculated after importing the latitude and longitude values from the ecological database into the GMPGIS. Finally, the global potential distribution and ecological factor values of the four endangered plants were obtained (figure7). The results were verified by MaxEnt.


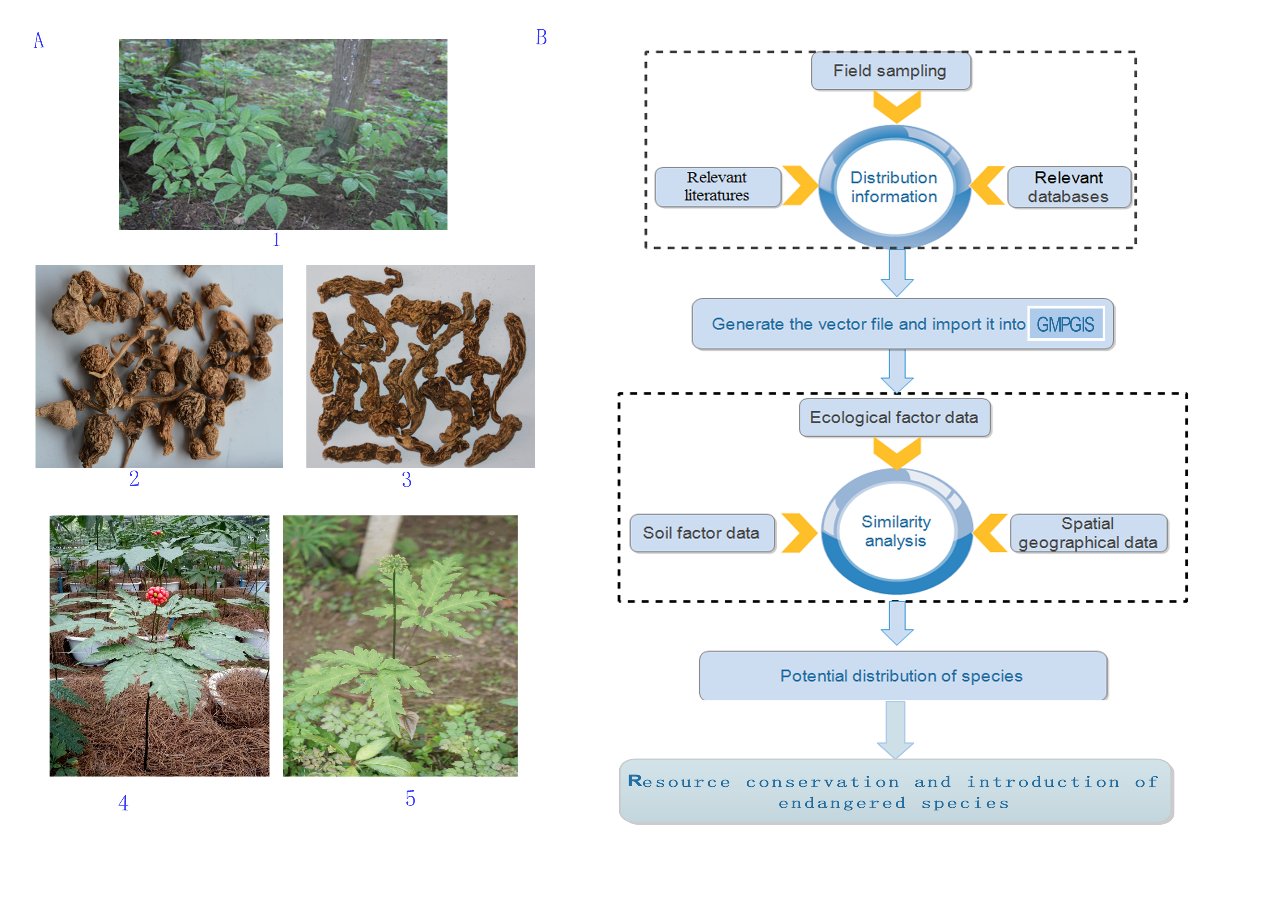


**Figure7:** Picture of plant and materials (A) (1: *P.japonicus*, 2*,* 5: *P. japonicus var. major*, 3: *P. zingiberensis*，4:*P. stipuleanatus*), and flow-chart of potential distribution similarity analysis (B).

4.1 Data collection

Data on the distributions of *P. japonicus, P. japonicus var. major, P. zingiberensis*, and *P. stipuleanatus* were collected from our field sampling results combined with the data from the Chinese Virtual Herbarium (CVH), National Specimen Information Infrastructure (NSII), and Global Biodiversity Information Facility (GBIF). A total of 176 sampling points for *P. japonicus*, 274 points for *P. japonicus var. major*, 102 points for P. zingiberensis, and 101 points for P. stipuleanatus were collected and analyzed for the global ecological suitability.

4.2 GMPGIS database

The ecological database is important for the ecological suitability analysis. Climate data were mainly obtained from global climate data ([WorldClim](http://www.worldclim.org/)) and global climatologies for bioclimatic modeling (CliMond). WorldClim is recognized by the international community as a regional and accurate climate database and was established by Robert J. Hijmans[40], Susan Cameron, and Juan Parra of the Museum of Vertebrate Zoology at the University of California, Berkeley, United States. CliMond aims to share different formats of environmental data, modeling tools, and meteorological expertise for ecological studies, such as species distribution models, species endangerment models, and global climate change. This database provides data such as the monthly minimum temperature, monthly maximum temperature, monthly precipitation, monthly average, 9 a.m. relative humidity, monthly average, 3 p.m. relative humidity, and 35 bioclimatic data layers.

Soil data were mainly obtained from the Harmonized World Soil Database, which is jointly formed by the United Nations Educational, Scientific, and Cultural Organization and the International Institute for Applied Systems Analysis. These institutions provide data, such as soil name, texture, effective water content, organic matter, pH, conductivity, and other indicators. The soil types include Andosols, Acrisols, Alisols, Arenosols, Anthrosols, Chernozems, Calcisols, Cambisols, Fluvisols, Greyzems, Ferralsols, Regosols, Solonchaks, Solonetz, Gleysols, Gypsisols, Histosols, Leptosols, Kastanozems, Luvisols, Lixisols, Phaeozems, Planosols, Plinthosols, Nitisols, Podzoluvisols, Podzols, and Vertisols.

The model selected seven ecological factors, including the mean temperature of the coldest quarter, annual mean temperature, annual precipitation, annual average radiation, annual humidity, and soil type for the analysis of these plants.

4.3 Analysis by GMPGIS

The GMPGIS aim to predict the distribution of medicinal plants. Firstly, to eliminate the influence of a different method, the processing of ecological factors must be standardized. Secondly, to improve the k-means, a range-based technique is adopted to evaluate the ecological suitability models and to make them more adaptable to potential distribution prediction. Thirdly, the clustering layer is 13 reclassified, and the potential distribution of areas is discovered. Fourthly, the suitable soil layer are intersected with the climatic factors in a Euclidean distance layer. The main steps of the system are as follows：

(1) Step 1: Linear normalization is performed on the original data. Suppose that *min_A_* and *max_A_* were the minimum and maximum values of a layer A. Linear normalization maps a value *V_i_* of A to *V_i_* in the range [*newmin_A_, newmax_A_*] by computing the following:


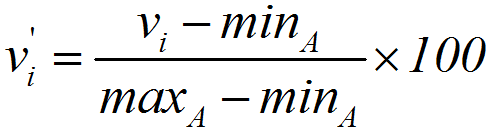
 (a)

(2) In our study, an improved k-means was adopted to evaluate the ecological suitability models. A range-based partitioning technique uses the critical size of a cluster *Di* to represent that cluster. Conceptually, the critical of a cluster is its marginal value *di*. The range can be defined in various ways, such as by the polyhedron assigned to the cluster. The difference between an object *p*∈*Di* and *di,* the representative of the cluster, is measured by dist (*p*, *di*), where dist (*x, y*) is the Euclidean distance between two points, *x* and *y*. The quality of cluster *Di* can be measured by the within-cluster variation, which is the sum of the squared error between all points in *Di* and the range *di*, defined as follows:


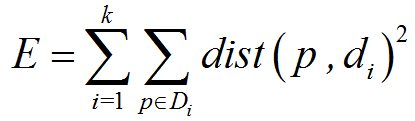
 (b)

Where


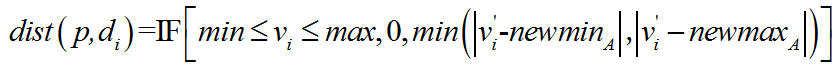
 (c)

where E is the sum of the *squared error* for all points in the data set, *p* is the point in space representing a given object, and *di* is the range of cluster *Di* (both *p* and *di* were multidimensional). *newmin_A_* is the minimum value after standardizing the layer; *newmax_A_* is the maximum value after standardizing the layer.

Step 3: According to the results of the distance calculation [*Min_d_*, *Max_d_*], the grid was classified, and the most similar ecological area was discovered.

Step 4: The suitable soil layer and climatic factors in the Euclidean distance layer were intersected.

4.4 Analysis by MaxEnt

The data of sampling points and environment variables were imported into the MaxEnt software. Parameter settings were as follows: the training set is 75% of the sampling point data, the test set is the remaining 25% used to examine the predictive ability of the model, and the jackknife was used to test the weight. The threshold-independent receiver operating characteristic and area under the receiver operating characteristic curve values were calculated by MaxEnt. The results were analyzed by ArcGIS 10.2, and the maps of ecologically suitable regions for endangered plants were obtained based on the main ecological factors.

**5. Conclusion**

The growth of endangered medicinal plants requires appropriate ecological conditions. Their quality are closely related to ecological factors such as climate and soil. The suitability distribution analysis of medicinal plants has been based on the traditional experience, single ecological factor, and single origin studies. Therefore, this analysis shows low efficiency and poor accuracy. GMPGIS with powerful spatial data management and analysis and mapping ability can combine the attributed and graphic data. The prediction of potential suitable distributions is conducive to the conservation of biological diversity. The results of the present analysis include output maps, charts, and other forms, which reduce the statistical and mapping work and achieve the visualization of regionalization for these *panax* species. Hidden information from the data can be revealed by the suitability analysis of producing areas from the geographic information system for Chinese traditional medicines. This study greatly improves the management level and application value of the resource information for traditional Chinese medicines, and provides a reference for resource conservation, introduction, and cultivation of endangered *Panax* species. This study just for the protection of species, and the scientific research, not open to other harmful behavior.

**Acknowledgments**: The work was supported by grants from the National Natural Science Foundation of China (No. 81473315, No.81274013), CAMS Innovation Fund for Medical Sciences (CIFMS) (No. 2016-I2M-3-015)，The Key Projects in the National Science and Technology Pillar Program (NO. 2011BAI07B08).

**Author Contributions:** Linfang Huang, Zhixia Du conceived and Jie Wu designed the study. Zhixia Du and Jie Wu processed the data, performed the analyses and analyzed the results, and wrote the manuscript. Linfang Huang, Zhixia Du, Jie Wu, Xiangxiao Meng, Jinhua Li and edited the manuscript. All authors read and approved the final version of the manuscript.

**Conflicts of Interest:** The authors declare no conflict of interest.

**Reference**

1. Yang, X.; Wang, R.; Zhang, S.; Zhu, W.; Tang, J.; Liu, J.; Chen, P.; Zhang, D.; Ye, W.; Zheng, Y., Polysaccharides from Panax japonicus C.A. Meyer and their antioxidant activities. *Carbohydrate Polymers* **2014,** 101, (1), 386-391.

2. Rai, A.; Yamazaki, M.; Takahashi, H.; Nakamura, M.; Kojoma, M.; Suzuki, H.; Saito, K., RNA-seq Transcriptome Analysis ofPanax japonicus, and Its Comparison with OtherPanaxSpecies to Identify Potential Genes Involved in the Saponins Biosynthesis. *Frontiers in Plant Science* **2016,** 7, (e0144).

3. Zhang, S.; Wang, R.; Zeng, W.; Zhu, W.; Zhang, X.; Wu, C.; Song, J.; Zheng, Y.; Chen, P., Resource investigation of traditional medicinal plant Panax japonicus (T.Nees) C.A. Mey and its varieties in China. *Journal of Ethnopharmacology* **2015,** 166, 79-85.

4. Shi, F. X.; Li, M. R.; Li, Y. L.; Jiang, P.; Zhang, C.; Pan, Y. Z.; Liu, B.; Xiao, H. X.; Li, L. F., The impacts of polyploidy, geographic and ecological isolations on the diversification of Panax (Araliaceae). *Bmc Plant Biology* **2015,** 15, (1), 1-11.

5. Liu, J.; Liu, Y.; Wang, Y.; Abozeid, A.; Zu, Y. G.; Zhang, X. N.; Tang, Z. H., GC-MS Metabolomic Analysis to Reveal the Metabolites and Biological Pathways Involved in the Developmental Stages and Tissue Response of Panax ginseng. *Molecules* **2017,** 22, (3).

6. Liu, Z.; Wang, C. Z.; Zhu, X. Y.; Wan, J. Y.; Zhang, J.; Li, W.; Ruan, C. C.; Yuan, C. S., Dynamic Changes in Neutral and Acidic Ginsenosides with Different Cultivation Ages and Harvest Seasons: Identification of Chemical Characteristics for Panax ginseng Quality Control. *Molecules* **2017,** 22, (5).

7. Xia, P.; Bai, Z.; Liang, T.; Yang, D.; Liang, Z.; Yan, X.; Liu, Y., High-performance liquid chromatography based chemical fingerprint analysis and chemometric approaches for the identification and distinction of three endangered Panax plants in Southeast Asia. *Journal of Separation Science* **2016,** 39, (20), 3880-3888.

8. Pimm, S. L.; Jenkins, C. N.; Abell, R.; Brooks, T. M.; Gittleman, J. L.; Joppa, L. N.; Raven, P. H.; Roberts, C. M.; Sexton, J. O., The biodiversity of species and their rates of extinction, distribution, and protection. *Science* **2014,** 344, (6187), 1246752.

9. Herrick, K.A.; F. Huettmann;M. A Lindgren, A global model of avian influenza prediction in wild birds: the importance of northern regions. Veterinary Research **2017**,44(1).

10. Han,X.S.; Guo,Y.M.; Mi,C.R.; F.Huettmann., Machine Learning Model Analysis of Breeding Habitats for the Blacknecked Crane in Central AsianUplands under Anthropogenic Pressures.Scientific Reports **2017**,7(1).

11. Mi,C.R.; Zu,q.;He,L.,Climate change would enlarge suitable planting areas of sugarcanes in China. International Journal of Plant Production **2017**, 11(1).

12. Jiao,S.G.; F. Huettmann;G,Y.M,advanced long-term birdbanding and climate data mining in spring confirm passerine population declines forhe Northeast Chinese-Russian flyway. Global and Planetary Change **2016**.

13. Guo,Y.M.; F. Huettmann;G,Y.M., Climate envelope predictions indicatean enlarged suitable wintering distribution for Great Bustards (Otis tarda dybowski) inChina for the 21st century. PeerJ **2016**,4(2).

14. Warren, D. L.; Seifert, S. N., Ecological niche modeling in Maxent: the importance of model complexity and the performance of model selection criteria. *Ecological Applications A Publication of the Ecological Society of America* **2011,** 21, (2), 335-42.

15. Mi, C., Why choose Random Forest to predict rare species distribution with few samples in large undersampled areas? Three Asian crane species models provide supporting evidence. *Peerj* **2017,** 5, (6).

16. Kalkvik, H. M.; Parkinson, C. L., Investigating niche and lineage diversification in widely distributed taxa: phylogeography and ecological niche modeling of the Peromyscus maniculatus species group. *Ecography* **2012,** 35, (1), 54-64.

17. Meng, X. X.; Huang, L. F.; Dong, L. L.; Xi-Wen, L. I.; Wei, F. G.; Chen, Z. J.; Jie, W. U.; Sun, C. Z.; Yu-Qi, Y. U.; Chen, S. L., Analysis of global ecology of Panax notoginseng in suitability and quality. *Acta Pharmaceutica Sinica* **2016**.

18. Liang, S.; Jie, W. U.; Xi-Wen, L. I.; Jiang, X. U.; Dong, L. L.; Sang, M. C.; Sun, C. Z.; Fujihara, N.; Chen, S. L., A study of global ecological adaptability and field selection practices of Panax ginseng. *China Journal of Chinese Materia Medica* **2016**.

19. Sánchez‐Fernández, D.; Lobo, J. M., Species distribution models that do not incorporate global data misrepresent potential distributions: a case study using Iberian diving beetles. *Diversity & Distributions* **2015,** 17, (1), 163-171.

20. Nag, C.; Chetan, N.; Karanth, K. P.; Praveen, K. K.; Gururaja, K. V.; Vasudeva, G. K., Delineating ecological boundaries of Hanuman langur species complex in peninsular India using MaxEnt modeling approach. *Plos One* **2014,** 9, (2), e87804.

21. Dan, L. W.; Seifert, S. N., Warren DL, Seifert SN. Ecological niche modelling in MaxEnt: the importance of model complexity and the performance of model selection criteria. Ecological Applications. *Ecological Applications* **2011,** 21, (2), 335-342.

22. Williams, H. W.; Cross, D. E.; Crump, H. L.; Drost, C. J.; Thomas, C. J., Climate suitability for European ticks: assessing species distribution models against null models and projection under AR5 climate. *Parasites & Vectors* **2015,** 8, (1), 1-15.

23. Gormley, A. M.; Woodford, L., Using presence‐only and presence–absence data to estimate the current and potential distributions of established invasive species. *Journal of Applied Ecology* **2011,** 48, (1), 25–34.

24. Anderson, R. P., A framework for using niche models to estimate impacts of climate change on species distributions. *Annals of the New York Academy of Sciences* **2013,** 1297, (1), 8-28.

25. Guisan, A.; Tingley, R.; Baumgartner, J. B.; Naujokaitislewis, I.; Sutcliffe, P. R.; Tulloch, A. I.; Regan, T. J.; Brotons, L.; Mcdonaldmadden, E.; Mantykapringle, C., Predicting species distributions for conservation decisions. *Ecology Letters* **2013,** 16, (12), 1424-35.

26. Folmer, E. O.; Olff, H.; Piersma, T., How well do food distributions predict spatial distributions of shorebirds with different degrees of self-organization? *Journal of Animal Ecology* **2010,** 79, (4), 747-756.

27. Moat, J.; Williams, J.; Baena, S.; Wilkinson, T.; Gole, T. W.; Challa, Z. K.; Demissew, S.; Davis, A. P., Resilience potential of the Ethiopian coffee sector under climate change. **2017**.

28. Elith, J.; Phillips, S. J.; Hastie, T.; Dudík, M.; Chee, Y. E.; Yates, C. J., A statistical explanation of MaxEnt for ecologists. *Diversity & Distributions* **2011,** 17, (1), 43-57.

29. Wang, E.H.;Liu, S.L.;Song, Z., Study Progression About Medicinal Plant Cold-resistance of Physiological. [Chinese Agricultural Science Bulletin](http://xueshu.baidu.com/usercenter/data/journal?cmd=jump&wd=journaluri%3A%2891aff42644afc8b1%29%20%E3%80%8AChinese%20Agricultural%20Science%20Bulletin%E3%80%8B&tn=SE_baiduxueshu_c1gjeupa&ie=utf-8&sc_f_para=sc_hilight%3Dpublish&sort=sc_cited) **2014**, 30 (16) :79-84.

30. Hou,Y.;Ma, Y.;Zou, Li-si, The Effcts of ecological factors on the secondary metabolites in medicinal plants and their research methods. [Lishizhen Medicine & Materia Medica Research](http://xueshu.baidu.com/usercenter/data/journal?cmd=jump&wd=journaluri%3A%28110c5b176bfbed4a%29%20%E3%80%8ALishizhen%20Medicine%20%26%20Materia%20Medica%20Research%E3%80%8B&tn=SE_baiduxueshu_c1gjeupa&ie=utf-8&sc_f_para=sc_hilight%3Dpublish&sort=sc_cited) **2015.**

31. Xie,C.X.;Suo,F.M.;Jia,G.L., Correlation between ecological factors and ginsenosides.[Acta Ecologica Sinica](http://xueshu.baidu.com/usercenter/data/journal?cmd=jump&wd=journaluri%3A%2880a032b68659f92a%29%20%E3%80%8AActa%20Ecologica%20Sinica%E3%80%8B&tn=SE_baiduxueshu_c1gjeupa&ie=utf-8&sc_f_para=sc_hilight%3Dpublish&sort=sc_cited) **2011**

32. Jia,G-L.;Huang,L-F.;Suo,F-M., Correlation between ginsenoside contents in Panax ginseng roots and ecological factors, and ecological division of ginseng plantation in China. [Chinese Journal of Plant Ecology](http://xueshu.baidu.com/usercenter/data/journal?cmd=jump&wd=journaluri%3A%289d667572af26dabf%29%20%E3%80%8AChinese%20Journal%20of%20Plant%20Ecology%E3%80%8B&tn=SE_baiduxueshu_c1gjeupa&ie=utf-8&sc_f_para=sc_hilight%3Dpublish&sort=sc_cited)**2012** , 36 (4) :302-312.

33. Han,Z.M.;Zhao,S.J.;Liu,C.J., Effects of shading on growth and quality of triennial Clematis manshurica Rupr. [Acta Ecologica Sinica](http://xueshu.baidu.com/usercenter/data/journal?cmd=jump&wd=journaluri%3A%2880a032b68659f92a%29%20%E3%80%8AActa%20Ecologica%20Sinica%E3%80%8B&tn=SE_baiduxueshu_c1gjeupa&ie=utf-8&sc_f_para=sc_hilight%3Dpublish&sort=sc_cited)**2011** , 31 (20) :6005-6012.

34. Zuang,W.J.;Yu,R.C.;Zhou,T.J., Spatial Distribution and Temporal Variation of Soil Moisture over China Part II: The Evaluations for Coupled Models' Simulations.[Chinese Journal of Atmospheric Sciences](http://xueshu.baidu.com/usercenter/data/journal?cmd=jump&wd=journaluri%3A%284e014c190c8d398%29%20%E3%80%8AChinese%20Journal%20of%20Atmospheric%20Sciences%E3%80%8B&tn=SE_baiduxueshu_c1gjeupa&ie=utf-8&sc_f_para=sc_hilight%3Dpublish&sort=sc_cited) **2008** , 32 (5) :1128-1146.

35. Lu,S.P.;Sui,X-X.;Sun,Q., Biological Functions of Secondary Metabolism of Medicinal Plants and Influences of Ecological Environment.[Natural Product Research & Development.](http://xueshu.baidu.com/usercenter/data/journal?cmd=jump&wd=journaluri%3A%28c54943d6b70b5a65%29%20%E3%80%8ANatural%20Product%20Research%20%26%20Development%E3%80%8B&tn=SE_baiduxueshu_c1gjeupa&ie=utf-8&sc_f_para=sc_hilight%3Dpublish&sort=sc_cited)**2006** , 18 (6) :1027-1032.

36. Ma,R-J.;Jiang,Z-G., Impact of global climate change on wildlife.[Acta Ecologica Sinica](http://xueshu.baidu.com/usercenter/data/journal?cmd=jump&wd=journaluri%3A%2880a032b68659f92a%29%20%E3%80%8AActa%20Ecologica%20Sinica%E3%80%8B&tn=SE_baiduxueshu_c1gjeupa&ie=utf-8&sc_f_para=sc_hilight%3Dpublish&sort=sc_cited). **2005**, 25 (11) :3061-3066.

37. Zou,H.Y.;Zhou,D.;Zhao,H., Determination of Chikusetsusaponin Ⅴ and Ⅳ a in Panax Japonicus by RP-HPLC. Journal of Liaoning University of Traditional Chinese Medicine. **2015**

38. Wu,J.;Tang,H.;Huang,L.F., Research and analysis of globally ecological suitability for Taxus plants. Acta Pharmaceutica Sinica. **2017**.

39. Chen S L, Xiang L, Li L, et al. Global strategy and raw material production on artemisinin resources regeneration (in Chinese). Chin Sci Bull. **2017**, 62: 1982–1996.

40. Hijmans, R.; Cameron, S.; Parra, J.; Jones, P., WorldClim—global climate data. **2005**.

**Sample Availability:** Samples of the compounds ...... are available from the authors.

© 2017 by the authors. Submitted for possible open access publication under the
terms and conditions of the Creative Commons Attribution (CC BY) license (http://creativecommons.org/licenses/by/4.0/).
