# Supplementary material for: Predicting the Global Potential Distribution of Four Endangered Panax Species in Middle-and Low-Latitude Regions of China by the Geographic Information System for Global Medicinal Plants (GMPGIS)
Source: Molecules. 2017 Sep 28;22(10):1630. doi: 10.3390/molecules22101630 (PMC6151741; doi:10.3390/molecules22101630)
Supplement: Supplementary File 1 [file molecules-22-01630-s001.zip › responses to reviewers’commens/Firet response to revises/response to revises-1.docx]

Dear Editor:

We greatly appreciate the referees and your efforts in handling our manuscript. We thank you for your favorable consideration and the referees’ insightful comments.

We already carefully read the comments from the referees and revised the manuscript according to their suggestions. All these changes have been marked in red color. We are sure it has been greatly improved. And we hope that the revised version is acceptable for publication in molecules.

The responses to the referee’s comments point by point are listed as below. Please feel free to contact us with any questions and we are looking forward to your consideration.

Thanking you most sincerely for your time and consideration.

Referee(s)' Comments to Author:

**Reviewer: 1**

**1.Conceptually, GMBGIS is a good idea, but it’s not written in stone, hardly relevant or precise. How is really using it? As described, it’s more like a ‘homebrew’ and exploiting/using the Maxent prediction with some ‘basement coding’ and linked with odd understandings of statistics and for trying best predictions (which are not achieved, btw). This approach is widely insufficient for several reasons: a) the entire operation looks pretty a copy-cat approach, b) it’s a black box (presumably an automated code (which one ? Is R involved ?) just linking steps together), c) it uses old and outdated concepts (=NO relevant Data Mining and Machine Learning), and it is based on JUST predictors (=bias; we have 104 now, and why not using them ?).**

**Response:** Our goal is to protect endangered medicinal plants and benefits mankind by establishing the models of predict the distribution and analysis ecological factors.

We may be not clearly stated in previous manuscript, and we have concretely expounded in present article, please see line 276-306. The GMPGIS model was created by the Institute of Chinese Materia Medica to predict the distribution of medicinal plants. The main inventor belong to our research group，and we also got an China patent and published a book named“Ecological suitability distribution of Chinese medicinal herb”published in Sciences Press. Please refer to fig1. and fig2.

Figure1: Authorization patent of GMPGIS.

Figure2: Ecological suitability distribution of Chinese medicinal herb

In our previous study, the two models—GMPGIS and MaxEnt—have been compared by methodology analysis, take *Andrographis paniculata (Burm. F.) Nees, Panax quinquefolium* and *Crocus sativus*as example. From the quantitative evaluations, we can see the AUC values and the omissionrates of the GMPGIS were superior to those of MAXENT. However, the article have not been published, we only provide a table about this precision. Please refer to table 1.

**2. Please understand and follow Leo Breiman (2001; Two Cultures). It’s a must: inference throughpredictions! This is the only way how things can move forward.**

**Response:** We really appreciate your comment. We have already corrected and added related literatures about results and discussion to support the hypothesis. The GMPGIS model was designed based on statistics, ecology, botany, etc.

We have done some studies by using GMPGIS to guide the introduction and production of *Panax ginseng C. A. Mey* and *Panax notoginseng (Burk.)F. H. Chen* in China, and the reliability of the system has been verified successfully. Please see the some of my papers, monograph and patent.

Huang Linfang, Duan Baozhong, Chen Shilin et al. Mapping the potential distribution of high artemisinin-yielding Artemisia annua L. (Qinghao) in China with a Geographic Information System Chinese Medicine[J] Chinese Medicine,2010;5(1):1-8.

Meng, X, Huang L. & Chen, S. Analysis of global ecology of Panax notoginseng in suitability and quality. Acta Pharmaceutica Sinica 51, 1483−1493 (2016).

Shen, L, Wu, J. & Chen, S. A study of global ecological adaptability and field selection practices of Panax ginseng. China journal of material medica 18, 3314-3322 (2016).

Wu, J, Tang H, Huang Lin-fang. Research and analysis of globally ecological suitability for Taxus plants. Acta Pharmaceutica Sinica 52, 1186−1195 (2017).

Chen S L, Xiang L, Li L, et al. Global strategy and raw material production on artemisinin resources regeneration (in Chinese). Chin Sci Bull,

2017, 62: 1982–1996, doi: 10.1360/N972017-00286.

we also got an China patent(certificate No.1253173) and published a book named“Ecological suitability distribution of Chinese medicinal herb”.

**3. Figure 2 needs to show confidence intervals (not quantiles or standard errors).**

**Response:** Thanks for your friendly reminder! The drawback of boxplot is that it does not provide about data distribution skewness and tail heavy degree of precise measurement. Therefore, the application of the boxplot is best combined with other descriptive statistical tools such as mean, standard deviation, skewness, distribution function to describe the distribution of the data set shape.

Figure2 is boxplot that about ecological factors, refers to roughly estimate symmetry, roughly observe dispersion degree. This figure 2 were used in the comparison of several samples, and observed the rough range of ecological factors. Therefore, this article does not have an accurate calculation such as confidence intervals, standard errors. Thank you again.

**4. Potential distribution: What is it, following what citation, e.g. Hutchinson, Grinnell , Eltonian ? see for instance book by Cushman and Huettmann 2010 or other citations.**

**Response:** Thanks for your advice, I have read a lot of literatures and recognized this concept deeply. And change it to Potential suitable distribution.

**5. No, Worldclim is 100% NOT very accurate, certainly not for Himalaya region, besides other regions. It has gaps there and many bugs. Widely known (only data mining can overcome it).**

**Response:** Thanks for your friendly reminder! Worldclim is not very accurate, most databases are just a reference. Therefore, this model have some screen .We compared precipitation data from 27 weather stations over a 30-year period (1984–2014) in GMPGIS against the WorldClim 34 data (1960–1990), found acceptable to gret agreement between the two data sets, when visualized as c. 30 year averages for numerous locations. Climate profiles (30-year period; 1984–2014) for total monthly rainfall and average monthly temperature (minimum, median and maximum) for an additional 20 weather stations (National Meteorology Agency) situated within, or adjacent to, plant growing areas, were also found to be in good agreement with 1960–1990 WorldClim 34 data.

**6. Data normality is not so precise for a procedure; pretty poor and when all is pushed in a scale 0-100. Why not keeping the units as they are ?**

**Response:**Thanks for your friendly reminder! We may be not clearly stated in previous manuscript .Data need to be standardized, for eliminate the influence of dimensional and put all factors in a range to clustering in GMPGIS model. The process is :(a) Linear normalization is performed on the original data, (b)an improved k-means was adopted to evaluate the ecological suitability models,(c)According to the results of the distance calculation [*Min_d_, Max_d_*], the grid was classified, and the most similar ecological area was discovered,(d)The suitable soil layer and climatic factors in the Euclidean distance layer were intersected.

I have written content more detailed about method part, I believe that readers will understand the model, please see line 276-306 in article.

**7. Similarity Cluster Analysis is done very poor and not needed. See Betts et al. It just makes predictions worse. What are the method details for this analysis step ?**

**Response:** Thanks for your friendly reminder! It's not clearly stated in previous manuscript, we have concretely expounded in present article. The model need Similarity Cluster Analysis. The process is :(a) Linear normalization is performed on the original data, (b)an improved k-means was adopted to evaluate the ecological suitability models,(c)According to the results of the distance calculation [*Min_d_, Max_d_*], the grid was classified, and the most similar ecological area was discovered,(d)The suitable soil layer and climatic factors in the Euclidean distance layer were intersected.

**8. add an ethics statement for this work and its predictions please, e.g. are the plants now more poached or protected ? From all I know about China, there is a large amount of poaching and no good enforcement while the commercial (capitalistic) pressure on the plant population is rising.My biggest concern is that this methods, data and predictions are NOT all open access and not freely available. They must be, for a transparent and repeatable science please. It cannot be done any other.**

**Response:** Thanks for your friendly reminder! I also thought about this issue, refer to your article, we have added the ethics statement for this study. This study just for the protection of species, and the scientific research, not open to other harmful behavior, please see line 329.

**9. Abstract, first phrase. Should read: “Nowadays, global biodiversity is…”What plants, and how really selected/defined ?**

**Response:**This sentence have been modified in the article, line 18-19 “Global biodiversity is strongly influenced by the decrease in endangered biological species”.

**10. The 7 climate predictors come cheap, and should be extended. We use 104 these days. Why not used ?**

**Response:** The GMPGIS model was established to predict the distribution of medicinal plants and provide a reference for plant’s protection and introduction. We early analyzed the biological characteristics of medicinal plants, found out the ecological factors closely related to medicinal plant growth and development and the accumulation of secondary metabolites, according to statistics, ecology, botany, and related literature and experience. Finally, selected seven related ecological factors for medicinal plants, include the mean temperature of the coldest quarter, annual mean temperature, annual precipitation, annual average radiation, annual humidity, and soil type for the analysis.

**11. Panax needs a Taxonomic Serial Number (TSN; see ITIS.GOV) and for all its** species. It gets very confusing any other.

**Response:** Thank you for providing the website, *Panax japonicus (T. Nees) C. A. Meyer*recorded Chinese Pharmacopeia and Japanese Pharmacopeia, Taxonomic Serial Number is No:822354.

There not TSN in IT IS.GOV about *Panax japonicus var. major (Burkill) C. Y. Wu & K. M. Feng, Panax zingiberensis C. Y. Wu & K. M. Feng* and *Panax stipuleanatus C. T. Tsai & K. M. Feng,* due to different language or other reasons. Therefore, we have provided some information ([www.theplantlist.org](http://www.theplantlist.org) and [www.tropicos.org](http://www.tropicos.org)):

*Panax japonicus var. major (Burkill) C. Y. Wu & K. M. Feng*

*Panax zingiberensis C. Y. Wu & K. M. Feng*

*Panax stipuleanatus C. T. Tsai & K. M. Feng*

*Panax japonicus var. major* recorded Chinese Pharmacopeia, is known as “the king of herbs” in Chinese folk.*Panax japonicus var. major (Burkill) C. Y. Wu & K. M. Feng, Panax zingiberensis C. Y. Wu & K. M. Feng and Panax stipuleanatus C. T. Tsai & K. M. Feng* are also widely used as traditional ethnic medicines.

**12. The data source is entirely unclear. Why not just using GBIF.org ? If the data are not there, it’s not transparent and repeatable research. Looks pretty locked up and shady to me.**

**Response:** I'm very sorry didn't write abbreviation in previous manuscript. We have mentioned Global Biodiversity Information Facility in previous manuscript, but did not give abbreviation. Now, I've added the abbreviation of “GBIF” in present article. Please see line 248 “the data from the Chinese Virtual Herbarium (CVH), National Specimen Information Infrastructure (NSII), and Global Biodiversity Information Facility (GBIF)”.

**13. The following statement was made many times by now: “…used GMPGIS to predict the global distribution of panax…”This concept looks prettyshady  “Repeated spatial clusters of localities were eliminated, and geographic221 coordinates were recorded for each distribution site on Google Earth”.It’s not needed nor correct.I propose authors run a VARCLUST (R hmisc) on their analysis to show correlations and what they look like.**

**Response:** I have rewritten content about method part more detailed, please see line 276-306 in article.

Thank you for all the articles the reviewer given, I have read it all, and as a references for our article.

Herrick, K.A.; F. Huettmann;M. A Lindgren, A global model of avian influenza prediction in wild birds: the importance of northern regions. Veterinary Research **2017**,44(1).

Han,X.S.; Guo,Y.M.; Mi,C.R.; F.Huettmann., Machine Learning Model Analysis of Breeding Habitats for the Blacknecked Crane in Central AsianUplands under Anthropogenic Pressures.Scientific Reports **2017**,7(1).

Mi,C.R.; Zu,q.;He,L.,Climate change would enlarge suitable planting areas of sugarcanes in China. International Journal of Plant Production **2017**, 11(1).

Jiao,S.G.; F. Huettmann;G,Y.M,advanced long-term birdbanding and climate data mining in spring confirm passerine population declines forhe Northeast Chinese-Russian flyway. Global and Planetary Change.**2016**.

Guo,Y.M.; F. Huettmann;G,Y.M., Climate envelope predictions indicatean enlarged suitable wintering distribution for Great Bustards (Otis tarda dybowski) inChina for the 21st century. PeerJ **2016**,4(2).

Mi, C., Why choose Random Forest to predict rare species distribution with few samples in large undersampled areas? Three Asian crane species models provide supporting evidence. *Peerj* **2017,** 5, (6).
