# Supplementary material for: Predicting the Global Potential Distribution of Four Endangered Panax Species in Middle-and Low-Latitude Regions of China by the Geographic Information System for Global Medicinal Plants (GMPGIS)
Source: Molecules. 2017 Sep 28;22(10):1630. doi: 10.3390/molecules22101630 (PMC6151741; doi:10.3390/molecules22101630)
Supplement: Supplementary File 1 [file molecules-22-01630-s001.zip › responses to reviewers’commens/Firet response to revises/response to revises-2.docx]

Dear Editor:

We greatly appreciate the referees and your efforts in handling our manuscript. We thank you for your favorable consideration and the referees’ insightful comments.

We already carefully read the comments from the referees and revised the manuscript according to their suggestions. All these changes have been marked in red color. We are sure it has been greatly improved. And we hope that the revised version is acceptable for publication in molecules.

The responses to the referee’s comments point by point are listed as below. Please feel free to contact us with any questions and we are looking forward to your consideration.

Thanking you most sincerely for your time and consideration.

Referee(s)' Comments to Author:

**Reviewer: 2**

**1.The font used for Table 1 is too small and difficult to read.**

**Response:** We have revised the Table 1, the reader can clearly read.

**Table1:**The range of ecological factors for four endangered panax species.

| Latin name of species | Annual mean temperature/℃ | Mean temperature of coldest quarter/℃ | Mean temperature of warmest quarter/℃ | annual precipitation/mm | annual humidity/% | annual average radiation/ w**^.^**m^-2^ |
| --- | --- | --- | --- | --- | --- | --- |
| P.japonicus | 2.6 ~22.3 | -7.0 ~ 14.3 | 10.7 ~ 28.8 | 539 ~2273 | 49.4 ~ 75.5 | 118.7~ 157.6 |
|  | soil types :Lixisols，Arenosols，Chernozems，Luvisols，Ferralsols，Acrisols，Andosols，etc. | | | | | |
| P.japonicus var.major | -8.6~20.5 | -17.5~14.7 | -1.9~26.7 | 272~2562 | 44.9~73.3 | 118.3~157.9 |
|  | soil types: Lixisols, Chernozems，Greyzems，Leptosols，Arenosols,etc. | | | | | |
| P.zingiberensis | 13~22.7 | 6.9~18.0 | 17.8~25.9 | 957~1772 | 54.1~75.3 | 136.3~160.1 |
|  | Types: Acrisols, Arenosols，Arenosols,etc. | | | | | |
| P.stipuleanatus | 15~20.6 | 8.8-14.9 | 19.7~24.9 | 888~2161 | 61.2~76.4 | 136.9~156.0 |
|  | soil types: Acrisols, Arenosols,etc. | | | | | |

**2. Most of the results section only shows the distribution and density map-plots of where the Panax species are distributed but the discussion mentions element of the results which are not supported in the findings/results.**

**Response:** Thanks for your advice! The analysis is inadequate in previous manuscript. We have rewritten the results and discussion, and added a number of literatures. Please see line 76-84 and line157-159.

**3. Line 148-149 “The present study shows that temperature is one of the important ecological factors”, the authors need to clearly show how this is an important ecological factor on each section in the findings in the results section. Table 2 show this element, but there is need for a few sentences to show exactly how temperature influence these plant species distribution, do high or low temperature reduce their distribution? What is the optimal temperature range for each Panax species studies?**

Response: Sentence have been modified, please see line 172-176 “The present study shows that the mean temperature of the coldest quarter is most important for these *panax* species.” and “in a certain scope of temperature lower temperature may lead to higher accumulation of ginsenosides for *panax* species”, according to contribution of ecological factor (table2 and related literatures), and the temperature range for each *Panax* species have been show in Table 1and Figure2.

Our previous studies shown that the temperature is most important ecological factor for the ginsenoside. Please refer to related literatures:

Xie,C.X.;Suo,F.M.;Jia,G.L., Correlation between ecological factors and ginsenosides.[Acta Ecologica Sinica.](http://xueshu.baidu.com/usercenter/data/journal?cmd=jump&wd=journaluri%3A%2880a032b68659f92a%29%20%E3%80%8AActa%20Ecologica%20Sinica%E3%80%8B&tn=SE_baiduxueshu_c1gjeupa&ie=utf-8&sc_f_para=sc_hilight%3Dpublish&sort=sc_cited) 2011

Jia,G-L.;Huang,L-F.;Suo,F-M., Correlation between ginsenoside contents in Panax ginseng roots and ecological factors, and ecological division of ginseng plantation in China. [Chinese Journal of Plant Ecology](http://xueshu.baidu.com/usercenter/data/journal?cmd=jump&wd=journaluri%3A%289d667572af26dabf%29%20%E3%80%8AChinese%20Journal%20of%20Plant%20Ecology%E3%80%8B&tn=SE_baiduxueshu_c1gjeupa&ie=utf-8&sc_f_para=sc_hilight%3Dpublish&sort=sc_cited) , 2012 , 36 (4) :302-312.

**4. Line 151-152 “The results of the present study show a strong correlation between ecological between ecological factors and cultivation”. I have tried to check for the correlation values and their level of significance in the results section. I did not find the correlation values their p-values. Can authors support this part with statistical findings?**

**Response:**Sentence have been modified, please see line 178-179“The results of the previous study show a strong correlation between ecological factors and cultivation”, and addedrelevant literature:

Han,Z.M.;Zhao,S.J.;Liu,C.J., Effects of shading on growth and quality of triennial Clematis manshurica Rupr. [Acta Ecologica Sinica](http://xueshu.baidu.com/usercenter/data/journal?cmd=jump&wd=journaluri%3A%2880a032b68659f92a%29%20%E3%80%8AActa%20Ecologica%20Sinica%E3%80%8B&tn=SE_baiduxueshu_c1gjeupa&ie=utf-8&sc_f_para=sc_hilight%3Dpublish&sort=sc_cited) , 2011 , 31 (20) :6005-6012.

Zuang,W.J.;Yu,R.C.;Zhou,T.J., Spatial Distribution and Temporal Variation of Soil Moisture over China Part II: The Evaluations for Coupled Models' Simulations.[Chinese Journal of Atmospheric Sciences](http://xueshu.baidu.com/usercenter/data/journal?cmd=jump&wd=journaluri%3A%284e014c190c8d398%29%20%E3%80%8AChinese%20Journal%20of%20Atmospheric%20Sciences%E3%80%8B&tn=SE_baiduxueshu_c1gjeupa&ie=utf-8&sc_f_para=sc_hilight%3Dpublish&sort=sc_cited), 2008 , 32 (5) :1128-1146.

**5. Line 199-200, The sentence that reads, “We studied and compared the difference between GMPGIS and MAxExt and found that GMPGIS shows higher precision than MaxEnt” this sentence is not supported with explicit findings in the Results Section. It would be good if the authors provide a comparative table showing the similarities and differences for the two methods in the Results Section.**

**Response:** The GMPGIS model was created by the Institute of Chinese Materia Medica to predict the distribution of medicinal plants. Our group have made a study, the two models—GMPGIS and MaxEnt—have been compared by methodology, take *Andrographis paniculata (Burm. F.) Nees, Panax quinquefolium*and *Crocus sativus*as example. From the quantitative evaluations, we can see the AUC values and the omissionrates of the GMPGIS were superior to those of MAXENT. However, the article have not been published, we only provide a figure about this precision for you. Please see table 1.

**6. Line 154-155, “The sentence reading “This study aims to predict the potential distribution of endangered …………..” should be moved to the introduction part, possibly just before the Results Section as part of the objectives. It does not fit well in the discussion.**

**Response:**Sentence had been moved to the introduction part, line 68-71.

**7. Methods Section – authors should only discuss their results, for example; Line 228-203 Sentence “WorldClim is recognized by ……………..” can be omitted because it is not part of the methodology.**

**Response:**This sentence had been omitted.

**8. In general, the Results Section should provide explicit findings that should support the discussion while the Discussion Section should be based on the findings in the Results Section with some support of literature review. The Methods Section should also be explicit only discussion the methods used in the study without any justifications as to whether the approach used is well recognized or not.**

**Response:**I have already corrected and added related literature in results and discussion.

We added related literatures:

Wang, E.H.;Liu, S.L.;Song, Z., Study Progression About Medicinal Plant Cold-resistance of Physiological. [Chinese Agricultural Science Bulletin](http://xueshu.baidu.com/usercenter/data/journal?cmd=jump&wd=journaluri%3A%2891aff42644afc8b1%29%20%E3%80%8AChinese%20Agricultural%20Science%20Bulletin%E3%80%8B&tn=SE_baiduxueshu_c1gjeupa&ie=utf-8&sc_f_para=sc_hilight%3Dpublish&sort=sc_cited), 2014, 30 (16) :79-84.

Hou,Y.;Ma, Y.;Zou, Li-si, The Effcts of ecological factors on the secondary metabolites in medicinal plants and theirresearch methods. [Lishizhen Medicine & Materia Medica Research](http://xueshu.baidu.com/usercenter/data/journal?cmd=jump&wd=journaluri%3A%28110c5b176bfbed4a%29%20%E3%80%8ALishizhen%20Medicine%20%26%20Materia%20Medica%20Research%E3%80%8B&tn=SE_baiduxueshu_c1gjeupa&ie=utf-8&sc_f_para=sc_hilight%3Dpublish&sort=sc_cited) . 2015.

Xie,C.X.;Suo,F.M.;Jia,G.L., Correlation between ecological factors and ginsenosides.[Acta Ecologica Sinica.](http://xueshu.baidu.com/usercenter/data/journal?cmd=jump&wd=journaluri%3A%2880a032b68659f92a%29%20%E3%80%8AActa%20Ecologica%20Sinica%E3%80%8B&tn=SE_baiduxueshu_c1gjeupa&ie=utf-8&sc_f_para=sc_hilight%3Dpublish&sort=sc_cited) 2011

Jia,G-L.;Huang,L-F.;Suo,F-M., Correlation between ginsenoside contents in Panax ginseng roots and ecological factors, and ecological division of ginseng plantation in China. [Chinese Journal of Plant Ecology](http://xueshu.baidu.com/usercenter/data/journal?cmd=jump&wd=journaluri%3A%289d667572af26dabf%29%20%E3%80%8AChinese%20Journal%20of%20Plant%20Ecology%E3%80%8B&tn=SE_baiduxueshu_c1gjeupa&ie=utf-8&sc_f_para=sc_hilight%3Dpublish&sort=sc_cited) , 2012 , 36 (4) :302-312.

Han,Z.M.;Zhao,S.J.;Liu,C.J., Effects of shading on growth and quality of triennial Clematis manshurica Rupr. [Acta Ecologica Sinica](http://xueshu.baidu.com/usercenter/data/journal?cmd=jump&wd=journaluri%3A%2880a032b68659f92a%29%20%E3%80%8AActa%20Ecologica%20Sinica%E3%80%8B&tn=SE_baiduxueshu_c1gjeupa&ie=utf-8&sc_f_para=sc_hilight%3Dpublish&sort=sc_cited) , 2011 , 31 (20) :6005-6012.

Zuang,W.J.;Yu,R.C.;Zhou,T.J., Spatial Distribution and Temporal Variation of Soil Moisture over China Part II: The Evaluations for Coupled Models' Simulations.[Chinese Journal of Atmospheric Sciences](http://xueshu.baidu.com/usercenter/data/journal?cmd=jump&wd=journaluri%3A%284e014c190c8d398%29%20%E3%80%8AChinese%20Journal%20of%20Atmospheric%20Sciences%E3%80%8B&tn=SE_baiduxueshu_c1gjeupa&ie=utf-8&sc_f_para=sc_hilight%3Dpublish&sort=sc_cited), 2008 , 32 (5) :1128-1146.

Lu,S.P.;Sui,X-X.;Sun,Q., Biological Functions of Secondary Metabolism of Medicinal Plants and Influences of Ecological Environment.[Natural Product Research & Development](http://xueshu.baidu.com/usercenter/data/journal?cmd=jump&wd=journaluri%3A%28c54943d6b70b5a65%29%20%E3%80%8ANatural%20Product%20Research%20%26%20Development%E3%80%8B&tn=SE_baiduxueshu_c1gjeupa&ie=utf-8&sc_f_para=sc_hilight%3Dpublish&sort=sc_cited) , 2006 , 18 (6) :1027-1032.

Ma,R-J.;Jiang,Z-G., Impact of global climate change on wildlife.[Acta Ecologica Sinica](http://xueshu.baidu.com/usercenter/data/journal?cmd=jump&wd=journaluri%3A%2880a032b68659f92a%29%20%E3%80%8AActa%20Ecologica%20Sinica%E3%80%8B&tn=SE_baiduxueshu_c1gjeupa&ie=utf-8&sc_f_para=sc_hilight%3Dpublish&sort=sc_cited), 2005 , 25 (11) :3061-3066.

Zou,H.Y.;Zhou,D.;Zhao,H., Determination of Chikusetsusaponin Ⅴ and Ⅳ a in Panax Japonicus by RP-HPLC. Journal of Liaoning University of Traditional Chinese Medicine.2015

Wu,J.;Tang,H.;Huang,L.F., Research and analysis of globally ecological suitability for Taxus plants. Acta Pharmaceutica Sinica.2017.

Chen S L, Xiang L, Li L, et al. Global strategy and raw material production on artemisinin resources regeneration (in Chinese). Chin Sci Bull,2017, 62: 1982–1996.
