# Supplementary material for: Predicting the Global Potential Distribution of Four Endangered Panax Species in Middle-and Low-Latitude Regions of China by the Geographic Information System for Global Medicinal Plants (GMPGIS)
Source: Molecules. 2017 Sep 28;22(10):1630. doi: 10.3390/molecules22101630 (PMC6151741; doi:10.3390/molecules22101630)
Supplement: Supplementary File 1 [file molecules-22-01630-s001.zip › responses to reviewers’commens/Second responde to revises/response to revises 1.docx]

Dear Editor:

We greatly appreciate your efforts in the handling of our manuscript. And we heartily thank you for your insightful comments. All comments help us clarify and improve the manuscript.

We have already carefully read the literature that was recommended by referee, and revised the manuscript according to referee’s suggestions. All of these changes have been marked in **red.** We are confident that these changes have greatly improved the quality of the paper, and we hope that the revised version is acceptable for publication in your Journal.

The responses to the referee’s comments point by point are listed as below. Please feel free to contact us with any questions.

I thank you most sincerely for your time and consideration.

Prof. Dr. Linfang Huang

The Institute of Medicinal Plant Development (IMPLAD)

lfhuang@implad.ac.cn

**Referee(s)' Comments to Author:**

**Reviewer: 1**

1.For instance, what software is the analysis and GMBGIS really in (version, name, code available, pseudo-code, IBM, MAC, 64 bit how to obtain it, metadata)? The associated book etc might be great, but we are not reading it, nor does the audience have it handy and available. We need basic details at least.

**Response:** We agree with the referee’s comments, the basic details of the GMPGIS is crucial. However, since this GMPGIS has been investigated in our lab, it is not available commercially so far. We aim to compare the novel GMPGIS with the Maxent, a typical tool for species distribution and environmental niche modeling. The GMPGIS is the first global evaluation of ecological availability for medicinal plants. It shows that deductive models can accurately predict species distribution globally. We conclude that the GMPGIS can play a significant role in advancing the protection and introduction of global medicinal plants.

With this GMPGIS, we published a number of articles and obtained the software copyright registration certificate, as well as one monograph. The specific information is as follows:

1) We have published some articles:

Chen S L, Xiang L, Li L, et al. Global strategy and raw material production on artemisinin resources regeneration (in Chinese). ***Chin Sci Bull,*** 2017, 62: 1982–1996, doi: 10.1360/N972017-00286

WU Jie, TANG Huan, HUANG Lin-fang. Research and analysis of globally ecological suitability for Taxus plants. Acta Pharmaceutica Sinica 2017, 52 (7): 1186−1195

MENG Xiang-xiao, HUANG Lin-fang, DONG Lin-lin. Analysis of global ecology of Panax notoginseng in suitability and quality. Acta Pharmaceutica Sinica 2016, 51 (9): 1483−1493

2) Software copyright registration number: 2016SR074556

3) We published a book: “Ecological suitability distribution of Chinese medicinal herb”published in Sciences Press, 2011 and 2017(two editions).

In the previous manuscript，we mentioned some steps of the GMPGIS briefly. Here, more basis details of **GMPGIS** (geographic information system for global medicinal plants) have been added in the article. The major steps are done by the ArcGis 10.2, please refer to Line 278-315 in article.

Ecologically suitable distribution maps originated in GMPGIS 10.4, which is a structure of C/S. The GADM database of Global Administrative Areas (2012, version 2.0) can be downloaded from www.gadm.org. We used a combination of the above two maps in ArcGIS 10.4(64bit)，which can be downloaded from [www.esri.com](http://www.esri.com).

2.Lacking TSNs are a nice finding, sure, but this means then...that these species as used are not all valid ?

ITIS is the global government authority on species, so what's going on ? Who is right and why ?

Are we modeling an artefact (ghost, ='yeti') ?

**Response:** There is not TSN in ITIS.GOV about *Panax japonicus var. major (Burkill) C. Y. Wu & K. M. Feng, Panax zingiberensis C. Y. Wu & K. M. Feng* and *Panax stipuleanatus C. T. Tsai & K. M. Fen*g. The names of these plants are legal but not appear on the ITIS.GOV due to different language or other reasons, such as be revised for synonym of other plants which as rejected Name in taxonomy. These plant names can be found in Flora Reipublicae Popularis Sinicae or the plant list (www.theplantlist.org), please see below:

1)*Panax japonicus var. major (Burkill) C. Y. Wu & K. M. Feng* has been reassigned as a synonym of *Panax pseudoginseng Wall. Var. japonicas (C. A. Mey.) Hoo &Tseng. Panax stipuleanatus C. T. Tsai & K. M. Feng* has been reassigned as a synonym of *Panax pseudoginseng Wall. Var.bipinnatifidus (seem.) Li.*

The Taxonomic Serial Number of *Panax pseudoginseng Wall* is No:506795.

(Flora Reipublicae Popularis Sinicae, vol. 54:180-188.Science Press, Beijing [In Chinese]).

2)*Panax zingiberensis C. Y. Wu & K. M. Feng* has been recorded in JOURNAL OF SYSTEMATICS AND EVOLUTION and be named by C. Y. Wu and K. M. Feng in 1975.

(Flora Reipublicae Popularis Sinicae, vol. 54:180-188.Science Press, Beijing [In Chinese]).

In the plant list (www.theplantlist.org), *Panax zingiberensis C.Y.Wu & Feng* is an accepted name, this name is the accepted name of a species in the genus Panax (family Araliaceae).The record derives from WCSP (data supplied on 2012-03-23) which reports it as an accepted name (record 146830) with original publication details: Acta Phytotax. Sin. 13(2): 42 1975.Full publication details for this name can be found in IPNI: http://ipni.org/urn:lsid:ipni.org:names:91594-1.

3.Much more detail is needed to explain all of that better.

I had expressed all of it in my review very clearly and with questions to be answered.

**Response:** Thanks for reviewer, every opinion of your is helpful, we took every single question pretty seriously，and answer them point by point. And thank you give us the chance, we will keep on learning and improve my articles.

4.Now, 7 predictors CAN NOT PREDICT WELL (impossible) and when 104 are available by now. It's a massive bias; must be (90% of data and information ignored)! They are not even vetted (varclust was suggested, WORLDCLIM has errors either way: how addressed?).

Authors are widely silent on those things. That's just not defendable.

**Response:** The GMPGIS model can be quickly predicted the distribution of medicinal plants. Currently, the GMPGIS selected 7 key ecological factors (especially related to its growth and accumulation of secondary metabolism) for medicinal plants according to biological characteristics of medicinal plants, statistics, ecology, botany, and related literatures, experience. With this GMPGIS, we published a number of articles and obtained the software copyright registration certificate, as well as one monograph. The 7 ecological factors include the mean temperature of the coldest quarter, annual mean temperature, annual precipitation, annual average radiation, annual humidity, and soil type.

These ecological factors are representative and comprehensive that reflects the three climate factor, such as light, heat, water and soil environmental factors. With these crucial ecological factors, we published a number of articles. The related literatures are as follows:

(1) The author reduced the19 BIOCLIM variables to nine that reflects the three climate factor, such as light, heat, water, for use in modelling. The same variables were used for the east and west split, to allow our models to be comparable. Using a modelling approach in combination with remote sensing, supported by rigorous ground-truthing, we project changes in suitability for coffee farming under various climate change scenarios, specifically by assessing the exposure of coffee farming to future climatic shifts.

(Justin Moat, Jenny Williams, Susana Baena. Resilience potential of the Ethiopian coffee sector under climate change. ***Nature Plants*.**2017)

(2)**The GMPGIS** was used to predict the distribution of high quality *Artemisia annua L.* Artemisinin renewable resource and raw material to produce lay the foundation for the "Belt and Road" strategy implementation for Chinese medicine resources countries around the world.

(Chen S L, Xiang L, Li L, et al. Global strategy and raw material production on artemisinin resources regeneration (in Chinese). ***Chin Sci Bull*,**

2017, 62: 1982–1996, doi: 10.1360/N972017-00286)

(3)In the article about map the climatic suitable habitat of oriental arborvitae，the author think that an excess of climatic variables can cause overfitting, so they selected 13 of the 23 climatic variables related to temperature, precipitation, growing degree days, thermal and moisture factors. **Temperature variables include annual mean temperature (AMT), max temperature of the warmest month (MTWM), min temperature of the coldest month (MTCM), and annual range of temperature (ART = MTWM-MTCM)**. Precipitation variables include **annual precipitation (AP)**, precipitation of wettest month (PWM), precipitation of driest month (PDM), and precipitation of seasonality (PSD = Monthly coefficient of variation of precipitation).

(Guoqing, Li.,sheng,Du., Mapping the climatic suitable habitat of oriental arborvitae (Platycladus orientalis) for introduction and cultivation at a global scale. ***Sci Rep*** 2016)

(4) In the article about Global alterations in areas of suitability for maize production from climate change and using a mechanistic species distribution model (CLIMEX), CLIMEX models the mechanisms affecting species and matches the geographic occurrences with meteorological data. **The five ecological factor variables request monthly climatic data variables include average minimum temperature (Tmin), average maximum temperature (Tmax), average precipitation (Ptotal), and relative humidity at 09:00 and 15:00 hours (RH09:00 and RH15:00) for the specific research locations**.

(NYZ ramirezcabral, L Kumar. Global alterations in areas of suitability for maize production from climate change and using a mechanistic species distribution model (CLIMEX). ***Sci Rep.****2017)*

(5)In the previous study, a significant positive correlation existed between soil moisture and ginsenosides contents ( Rb3 excluded ).The correlation between ginsenosides contents and climatic factors(annual active accumulated temperature，annual mean temperature July maximum temperature，July mean temperature，January minimum temperature，and January mean temperature) was strongly negative. In particular，there existed a significant negative correlation ( r ＞0. 6 ) between climatic factors and the ginsenosides Rg1，Re and Rb1，which were specified in the pharmacopoeia．

(Xie,C.X.;Suo,F.M.;Jia,G.L., Correlation between ecological factors and ginsenosides. [***Acta Ecologica Sinica***](http://xueshu.baidu.com/usercenter/data/journal?cmd=jump&wd=journaluri%3A%2880a032b68659f92a%29%20%E3%80%8AActa%20Ecologica%20Sinica%E3%80%8B&tn=SE_baiduxueshu_c1gjeupa&ie=utf-8&sc_f_para=sc_hilight%3Dpublish&sort=sc_cited) 2011)

(6)The main factors which influence the accumulation of secondary metabolites of medicinal plants are **temperature, soil, moisture and sunlight.**

(Hou,Y.;Ma, Y.;Zou, Li-si, The Effcts of ecological factors on the secondary metabolites in medicinal plants and their research methods. [***Lishizhen Medicine & Materia Medica Research***](http://xueshu.baidu.com/usercenter/data/journal?cmd=jump&wd=journaluri%3A%28110c5b176bfbed4a%29%20%E3%80%8ALishizhen%20Medicine%20%26%20Materia%20Medica%20Research%E3%80%8B&tn=SE_baiduxueshu_c1gjeupa&ie=utf-8&sc_f_para=sc_hilight%3Dpublish&sort=sc_cited)  2015.)

Thanks for suggestions! 104 ecological factors are available by now which may play a important role in the precise prediction. We would consider other model that add more relevant ecological factors to achieve a different purpose on further study.

5.The box plot argument of the authors is also widely wrong: what does Figure 2 show us ? Quantiles ? Confidence Intervals are indeed possible and simple to display. We need to know either way. Authors blanked here too.

**Response:** The box plot provides an intuitive range of ecological factors (Figure2), it play an important role in plant cultivation, and ecological factor scope of *P.japonicus* and *P. japonicus var. major* are much broader. The box plot be made by MATLAB, which show us the median, maximum，minimum, one-quarter, and three-quarters of ecological factors for four plants(“The MATLAB platform is optimized for solving engineering and scientific problems. The matrix-based MATLAB language is the world’s most natural way to express computational mathematics”).

Hence, we provide a Q-Q plot that is a scatter plot of the quantile and the quantile of the normal distribution. If all points are on a straight line, indicating that the sample conforms to the quantile of the state distribution, that is the normal distribution. Even if these points do not obey the normal distribution, it is still available in this experiment. Please see below:

FigureS1: The Q-Q plot of ecological factors, (A) mean temperature of coldest quarter,(B) annual humidity,(C) annual precipitation, (D)annual average radiation,(E) annual mean temperature,(F) mean temperature of warmest quarter.

And any suggest from you would be highly appreciated!
