# Supplementary material for: Predicting the Global Potential Distribution of Four Endangered Panax Species in Middle-and Low-Latitude Regions of China by the Geographic Information System for Global Medicinal Plants (GMPGIS)
Source: Molecules. 2017 Sep 28;22(10):1630. doi: 10.3390/molecules22101630 (PMC6151741; doi:10.3390/molecules22101630)
Supplement: Supplementary File 1 [file molecules-22-01630-s001.zip › responses to reviewers’commens/Second responde to revises/response to revises 2.docx]

Dear Editor:

We greatly appreciate your efforts in the handling of our manuscript. And we heartily thank you for your insightful comments. All comments help us clarify and improve the manuscript.

We have already carefully read the literature that was recommended by referee, and revised the manuscript according to referee’s suggestions. All of these changes have been marked in **red.** We are confident that these changes have greatly improved the quality of the paper, and we hope that the revised version is acceptable for publication in your Journal.

The responses to the referee’s comments point by point are listed as below. Please feel free to contact us with any questions.

I thank you most sincerely for your time and consideration.

Prof. Dr. Linfang Huang

The Institute of Medicinal Plant Development (IMPLAD)

lfhuang@implad.ac.cn

**Referee(s)' Comments to Author:**

**Reviewer: 2**

There a significant improvement of the manuscript compared to the first submission. Just a few minor corrections, lines 80-86 I think the sentence is too long. I suggest breaking it into two or more.Line 207,I suggest chaning the “prediction” to “predicted”.

**Response:** Yes, we have revised the text，please see line 83-87 and 209.
